# Seasonal Differences in Structural and Genetic Control of Digestibility in Perennial Ryegrass

**DOI:** 10.3389/fpls.2021.801145

**Published:** 2022-01-04

**Authors:** Vincent Colas, Philippe Barre, Frederik van Parijs, Lukas Wolters, Yannick Quitté, Tom Ruttink, Isabel Roldán-Ruiz, Abraham J. Escobar Gutiérrez, Hilde Muylle

**Affiliations:** ^1^Unité de Recherche Pluridisciplinaire Prairies et Plantes Fourragères (URP3F), National Research Institute for Agriculture, Food and Environment (INRAE), Lusignan, France; ^2^Plant Sciences Unit, Institute for Agricultural, Fisheries and Food Research (ILVO), Melle, Belgium; ^3^DSV zaden Nederland B.V., Ven Zelderheide, Netherlands; ^4^DSV France, Les Rosiers sur Loire, France; ^5^Department of Plant Biotechnology and Bioinformatics, Ghent University, Ghent, Belgium

**Keywords:** *Lolium perenne* L., quality, digestibility, season, cell wall, genotype, QTL

## Abstract

Perennial ryegrass is an important forage crop in dairy farming, either for grazing or haying purposes. To further optimise the forage use, this study focused on understanding forage digestibility in the two most important cuts of perennial ryegrass, the spring cut at heading and the autumn cut. In a highly diverse collection of 592 *Lolium perenne* genotypes, the organic matter digestibility (OMD) and underlying traits such as cell wall digestibility (NDFD) and cell wall components (cellulose, hemicellulose, and lignin) were investigated for 2 years. A high genotype × season interaction was found for OMD and NDFD, indicating differences in genetic control of these forage quality traits in spring versus autumn. OMD could be explained by both the quantity of cell wall content (NDF) and the quality of the cell wall content (NDFD). The variability in NDFD in spring was mainly explained by differences in hemicellulose. A 1% increase of the hemicellulose content in the cell wall (HC.NDF) resulted in an increase of 0.81% of NDFD. In autumn, it was mainly explained by the lignin content in the cell wall (ADL.NDF). A 0.1% decrease of ADL.NDF resulted in an increase of 0.41% of NDFD. The seasonal traits were highly heritable and showed a higher variation in autumn versus spring, indicating the potential to select for forage quality in the autumn cut. In a candidate gene association mapping approach, in which 503 genes involved in cell wall biogenesis, plant architecture, and phytohormone biosynthesis and signalling, identified significant quantitative trait loci (QTLs) which could explain from 29 to 52% of the phenotypic variance in the forage quality traits OMD and NDFD, with small effects of each marker taken individually (ranging from 1 to 7%). No identical QTLs were identified between seasons, but within a season, some QTLs were in common between digestibility traits and cell wall composition traits confirming the importance of hemicellulose concentration for spring digestibility and lignin concentration in NDF for autumn digestibility.

## Introduction

Forage grasses are the main sources of energy and protein for ruminants (Fick et al., 1994). The nutritive value of the forage has a significant impact on animal performance by influencing the intake rate, the volume of milk produced, and its quality (Thomas et al., 1981; Oba and Allen, 1999). For example, Casler and Vogel (1999) concluded in a study comprising five forage grass species, that an increase of 1% of dry matter digestibility [DMD, or the fraction of the dry matter (DM) that is digestible – closely linked to foraging nutritive value as discussed below] leads to a 3.2% increase of daily gain of beef cattle. In addition, Oba and Allen (1999) observed in a study that a 1% increase of fibre digestibility was associated with a 0.25 kg increase in 4% fat-corrected milk. Therefore, breeding for high nutritive value has become one of the main objectives in forage grasses, in addition to forage yield and disease resistance (Wilkins and Humphreys, 2003; Sampoux et al., 2011). This is, in particular, the case for perennial ryegrass (*Lolium perenne* L.), which is one of the most used forage grass species in temperate regions thanks to its good tolerance to grazing and its overall good nutritive quality (Wong, 2005; Semae, 2020).

The nutritive value of the forage is determined by the concentration of available energy (related to the concentration of digestible constituents) and the concentration of crude protein (Newman et al., 2006). The DM of grass forage consists of approximately 92.3% of an organic fraction (OM) and of a mineral fraction (7.7%), which is completely non-digestible. In the OM we can distinguish fibre constituents that are partially digestible, and non-fibre constituents that are almost completely digestible (Van Soest, 1967). Non-fibre constituents include water-soluble proteins and carbohydrates (WSC). Fibres include hemicellulose (HC), cellulose (C), and lignin in decreasing order of digestibility.

An improvement of the forage nutritive value can be achieved by increasing the proportion of non-fibre constituents and/or by increasing the digestibility of the fibre constituents (Buxton and Casler, 1993). Concerning the proportion of the different constituents, breeding efforts have resulted in an improvement of the forage nutritive value by decreasing the fibre content (Van Soest et al., 1991) or by increasing the WSC content (Humphreys, 1989). A higher amount of sugars in grass give improvements to cattle as larger intakes, increased milk production, less nitrogen excreted with urine, and more protein in milk (Miller et al., 2001; Parsons et al., 2011). In addition, WSC in forage promotes silage fermentation (McDonald and Henderson, 1964). However, a too high WSC content can raise subacute ruminal acidosis risk, or even to ruminal acidosis with high grain diets (O’Grady et al., 2008; González et al., 2012), and a too strong reduction of cell wall content can lead to a reduction of disease resistance for the plant (Smirnova and Kochetov, 2016). The protein content of grass forage is unequally partitioned between organs and is directly linked to nitrogen fertilisation and environmental conditions (Lemaire et al., 1984; Durand et al., 2010). Therefore, protein content has not been subjected to a strong selection up to now (Sampoux et al., 2011).

The composition of the cell walls has also a strong impact on the forage nutritive value. As Keys et al. (1969) demonstrated, the proportion of cellulose to HC influences the cell wall digestibility (and as a consequence also the DMD), since HC is more digestible than cellulose in grasses. However, the major factor influencing the digestibility of the fibre fraction is its lignin content (Woodman and Stewart, 1932; Jung and Vogel, 1986). Lignin is not only difficult to digest, but it also decreases cellulose and HC digestibility by decreasing their accessibility to enzymes through the creation of ether bonds (Grabber et al., 1997; Grabber, 2005; Bonawitz and Chapple, 2010). Furthermore, the lignin structure has also a strong influence on the cell wall digestibility (Grabber, 2005). The relationship between cell wall composition and cell wall digestibility has been largely documented in maize (Méchin et al., 2001; Barrière et al., 2003), but has got less attention in perennial forage grass species (Vetharaniam et al., 2014).

Several studies have shown that in forage grasses DMD depends on several factors including species, genotype within species, plant organ/tissue, organ/tissue age, season, and environmental conditions (Akin et al., 1987; Buxton and Casler, 1993; Groot et al., 1999; Bruinenberg et al., 2002; Carrère et al., 2010; Beecher et al., 2018). A large variability exists between forage grass species with DMD ranging from less than 60% in tall fescue to more than 80% in perennial ryegrass (Buxton and Casler, 1993; Pontes et al., 2007; Herbe-book, 2021). Genetic variability within species is also available, and in perennial ryegrass low to moderate heritabilities have been reported depending on the studied germplasm (Ullmann et al., 2017; Arojju et al., 2020). This diversity is exploited in breeding (Wilkins, 1997), and a progress of 0.39% DM per decade was observed for DMD during the period 1971–2004 in perennial ryegrass in Europe (Sampoux et al., 2011). This progress was realised with a 0.31 decrease in fibre content (% of DM) and a 0.55 increase in fibre digestibility (% of DM). Notably, this progress in forage quality was obtained simultaneously with progress in DM yield and disease resistance. It is also important to consider that the DMD of different plant parts is different and that the age of the plant organ has a major impact on its DMD (Buxton and Casler, 1993). Stems are less digestible than leaves, and the reduction of the DMD with ageing is more pronounced in stems than in leaves (Terry and Tilley, 1964; Wilman and Altimimi, 1982; Buxton and Casler, 1993). Therefore, DMD decreases strongly as the fraction of mature stems increases. This could explain the strong negative correlation between precocity and spring DMD across perennial ryegrass cultivars (Sampoux et al., 2011). To avoid problems with quality while maintaining sufficient DM yield, grass forage should be harvested just before heading. Similarly, it can be anticipated that genetic progress to avoid aftermath heading could contribute to an increase of DMD for forage harvested in summer and autumn. Also, seasonal differences have been reported in leaves, with a tendency for lower DMD under summer weather conditions (drought and high temperatures) (Pontes et al., 2007).

The genetic basis of DMD and more specifically the fibre content and its digestibility has been largely studied in species such as maize (Méchin et al., 2001; Riboulet et al., 2008; Lopez-Malvar et al., 2021), with some of the co-localising with candidate genes. In particular, genes involved in the biosynthesis of lignin have been associated with variation of DMD, and functional validation through transgenesis has been delivered for COMT (Catechol-*O*-methyltransferase) and CAD (Carbamoyl-Phosphate Synthetase 2, Aspartate Transcarbamylase, and Dihydroorotase) in maize and tall fescue (Chen et al., 2003, 2004; Hisano et al., 2009). In perennial ryegrass quantitative trait loci (QTLs) for DMD, fibre content and WSC have also been identified (Cogan et al., 2005). The 25 QTLs identified for WSC explained no more than 20% of the genetic variance suggesting a complex genetic determinism for this trait (Cogan et al., 2005; Turner et al., 2006; Shinozuka et al., 2012; Gallagher et al., 2015). To the best of our knowledge, only a few studies exist on the genetic determinism of fibre digestibility in perennial ryegrass.

The objective of this study was to obtain a better understanding of the variability of organic matter digestibility (OMD) in perennial ryegrass leaves harvested in spring and autumn on a wide collection of 580 genotypes. A large number of genotypes analysed allowed us to obtain interesting variations for all studied traits. A particular emphasis was placed on the variability of fibre digestibility and its link with the composition of fibre for a better comprehension of their links with the overall quality. We also investigated the genetic determinism of quality traits by a genome-wide association study (GWAS) approach based on candidate genes. The results obtained could help to improve breeding programs by focusing on major indicators for quality in spring and/or autumn.

## Materials and Methods

### Plant Material

The plant material used in this study consisted of 580 genotypes from diverse origins (Supplementary Table 1) chosen to represent a large genetic and phenotypic diversity (Veeckman et al., 2018). Among them, 42% were chosen among breeding material from ILVO, DSV, and Barenbrug, 22% represented commercial cultivars and 36% originated from wild accessions (of which 76% were of French origin).

### Trial Design

For each genotype, three clones were produced in trays by splitting tillers in September 2011 and 2012. The clones were moved to an unheated greenhouse (minimum temperature of 4°C) for vernalisation during the winter and then potted in 12 L containers in early March 2012 and 2013. Each plant consisted of three tillers, which were cut to the same height (*ca*. 4 cm). The soil in the pots was a combination of white peat and garden peat (30% DM, 20% OM), fertilised with 0.3 kg/m^3^ nitrogen phosphorus potassium (NPK) 14-16-18. The pots were transferred to a container field in open-air in Melle, Belgium (latitude 50°59′39″, longitude 3°47′5″ and 24 m above sea level) and arranged according to a randomised complete block design with three clonal replicates. The same setup was used for the 2 years. In 2012, the plants were fertilised with NPK (16-8-22) at the end of April, at the end of June, in the middle of July, and in early August (at a rate of 80, 30, 70, and 100 kg/ha, respectively). In 2013, the plants were fertilised with NPK (16-8-22) at the beginning of May, in the middle of June, in the middle of July, and in the middle of August (at a rate of 100, 30, 70, and 70 kg/ha, respectively). The pots were watered using drip irrigation once to twice a day, depending on irradiance and air temperature.

### Phenotyping

The plants were cut four times per year: once during the spring, twice during the summer, and once during the autumn. Only plant materials collected at the spring and autumn cuts were analysed. The sampling in spring and autumn differed slightly.

Each year, the spring cut was harvested on the actual day of the heading of each individual plant (i.e., when three ears were visible). This involved inspection of each individual plant every 2 or 3 days from the beginning of April to early July 2012 and from the beginning of May to the end of July 2013, to record the heading date of each individual plant. Plants were cut at 4 cm height in the time slot between 2:00 and 3:00 p.m. The biomass was dried in a ventilated oven for 48 h at 70°C, hand separated in the blade and “stem” (i.e., sheath and true stem together) fractions, and milled in a Fritsch cutting mill using a 0.5-mm sieve. In this study, we focused only on the blade samples which were harvested in both spring and autumn to allow comparison of the same organ between seasons. Plants that were harvested with too many spikes (weight proportion of spikes larger than 5%) and plants with extreme heading dates were not considered for further analysis. We considered the extreme 0.6% (i.e., 10 genotypes) of the distribution as “extreme heading dates”, which corresponded to >17 days before and >28 days after the median heading date in both years.

The summer cuts were performed on 9 July 2012, 6 August 2012, 8 July 2013, and 12 August 2013 to promote a good regrowth in September. On 7, 18, and 19 of September 2012 and on 17, 19, and 20 of September 2013, the autumn cuts were performed. Cuts were spread other 3 days because short time window (2:00 to 3:00 p.m). All plants were cut at 4 cm height, then dried and milled according to the same used protocol in spring. In this case, blades and sheaths were not separated because of the absence of stems.

### Wet Chemical Analysis and Near-Infrared Spectroscopy Prediction

All samples from spring and autumn cuts were scanned by near-infrared spectroscopy (NIRS) with FOSS XDS spectrometer (Foss, Hilleroed, Denmark). Wet-chemical analyses were performed on a selection of samples for seven traits: ADM expressed in percentage of fresh material, OM expressed as a percentage of DM, OMD, Neutral Detergent Fibre (NDF), Acid Detergent Lignin (ADL), Cellulose, and HC expressed in percentage of OM. Because of lacking data, some genotypes (20) were removed from the analysis. Only 550 were kept for further phenotypic analysis. The data for the 550 remaining genotypes are given in Supplementary Table 2.

The ADM was determined gravimetrically in duplicate by drying 2.5 g of the sample at 103°C for 24 h. This sub-sample was then ashed at 550°C, allowing calculation of OM as the proportion of ash-free ADM in ADM. OMD was determined following Goering and Van Soest (1970) *in vitro* rumen digestibility method, corrected for ashes.

The sequential van Soest method (Van Soest et al., 1991) was modified for use with the ANKOM Fibre Analyzer (ANKOM Technology Corporation, Fairpoint, NY, United States) to measure NDF, acid detergent fibre (ADF), and ADL. To determine NDF, 0.5 g of sample was brought in a filter bag and incubated in neutral detergent at 100°C for 1 h in the ANKOM. The residue was then washed with water at 100°C and acetone and dried at 103°C. NDF was calculated as the ash-free NDF residue (using the ash determined on the ADL fraction, see further), divided by the OM previously determined. ADF was determined by incubating the bags containing the NDF fraction in 5% H_2_SO_4_ and CTAB (20 g/L) for 1 h at 100°C in the ANKOM. The residue was then washed with water at 100°C and acetone, dried at 103°C, and weighed. ADF was calculated as the proportion of ash-free ADF residue divided by the OM previously determined. ADL was determined by submerging the bags containing the ADF fraction in a beaker with 72% H_2_SO_4_ for 3 h. Ash content was determined by heating the ADL residue to 550°C in a muffle furnace. ADL was calculated as the proportion of ash-free ADL residue divided by the OM previously determined. HC was calculated as NDF – ADF and cellulose as ADF – ADL in % OM.

A NIRS calibration was built with all the wet-chemistry data including spring and autumn samples of the 2 years (2012 and 2013). To assess the extent to which a trait could be predicted for samples not included in the calibration model, a leave-one-out cross-validation was performed.

### Derived Biochemical Data

Several traits were computed to study cell wall digestibility and its composition. Cell wall components (C, HC, and ADL) were expressed in percentage of NDF: C.NDF, HC.NDF, and ADL.NDF. Digestibility of NDF was computed using a formula derived from Struik (1983):


(1) $$\mathrm{NDFD}=100-100\times \frac{100-\mathrm{OMD}}{\mathrm{NDF}}$$

This formula is based on the assumption that all indigestible parts of the plant are contained in cell walls.

### Statistical Analysis of the Phenotypic Data

#### General Models and Heritability

The effect of genotype, year, season, and block was analysed using a mixed model with R package *lme4* (Bates et al., 2015) as follows:


(2) $$Y_{ijkl}=\mu +G_{i}+T_{j}+S_{k}+TS_{jk}+GT_{ij}+GS_{ik}+B_{l}\left(TS_{jk}\right)+e_{ijkl}$$

where *Y*_*ijkl*_ = the phenotype of genotype *i* (1,…, 550), in year *j* (2012, 2013), season k (spring, autumn) and block l (1, 2, and 3) (e.g., OMD, NDF, NDFD, C.NDF, HC.NDF, or ADL.NDF),

μ = overall mean,

*G*_*i*_ = random effect of genotype *i*, *G*_*i*_ ∼ *N*(0, *σ*^2^_*g*_),
*T*_*j*_ = fixed effect of year *j*,
*S*_*k*_ = fixed effect of season *k*,
TS*_*jk*_* = interaction of year *j* and season *k*,
GT*_*ij*_* = interaction of genotype *i* and year *j*, GT*_*ij*_* ∼ *N*(0,σ^2^*_*gt*_*),
GS*_*ik*_* = interaction of genotype *i* and season *k*, GS*_*ik*_* ∼ *N*(0,σ^2^*_*gs*_*),
B*_*l*_*(TS*_*jk*_*) = fixed effect of block *l* nested within year *j* and season *k*,
and *e*_*ijkl*_ = residual, assumed to be normally distributed, *e*_*ijkl*_ ∼ *N*(0,σ^2^*_*e*_*).

The data set was further analysed by season using the following model for spring and autumn separately:


(3) $$Y_{ijl}=\mu +G_{i}+T_{j}+GT_{ij}+B_{l}\left(T_{j}\right)+e_{ijl},$$

where *Y*_*ijl*_ = the phenotype of genotype *i* (1,…,550), in year *j* (2012, 2013) and bloc *l* (1, 2, and 3),

*B*_*l*_(*T*_*j*_) = fixed effect of block *l* nested within year *j*,
and *e*_*ijl*_ = residual, assumed to be normally distributed, *e*_*ijl*_ ∼ *N*(0,σ^2^*_*e*_*).

For each season, the broad-sense heritability (*H*^2^) for each trait was calculated as:


(4) $$\frac{\sigma_{G}^{2}}{\sigma_{G}^{2}+\sigma_{GT}^{2}+\sigma_{e}^{2}}.$$

For GWAS, Eq. 3 was used to calculate the Best Linear Unbiased Predictor (BLUP) of the genotype effect (μ + *G*_*i*_) per season over 2 years. Another model per season and per year was also used:


(5) $$Y_{il}=\mu +G_{i}+B_{l}+e_{il}$$

Where *Y*_*il*_ = the phenotype of genotype *i* (1,…,550) and bloc *l* (1,2,3),

*B*_*l*_ = fixed effect of block *l*,
and *e*_*il*_ = residual assumed to be normally distributed, *e*_*il*_ ∼ *N*(0,σ^2^*_*e*_*).

This model (Eq. 5) was used to calculate the BLUP of genotype effect (μ + *G*_*i*_) per season and per year.

#### Drivers of Organic Matter Digestibility and Neutral Detergent Fiber Digestibility

Linear models were built using the *lm* function in R (R Core Team, 2020). Two models were used: one with OMD as the dependent variable and NDF and NDFD as independent variables, and one with NDFD as dependent variable and HC.NDF and ADL.NDF as independent variables. We did not use C.NDF in the model as C.NDF + HC.NDF + ADL.NDF = 1 and because C.NDF is highly negatively correlated to HC.NDF (-0.96 for all years and seasons). Models were built for each season and each year separately and were compared.

#### Genetic Markers

The genetic markers reported in Veeckman et al. (2018) were used. These markers describe the sequence diversity in 503 candidate genes involved in plant development, plant architecture, phytohormone biosynthesis, and response pathways, and cell wall composition. This dataset consists of 252,406 SNPs and 5,074 indels identified in an association panel of 736 genotypes, including the 580 genotypes reported in this study (Veeckman et al., 2018). The reported markers are spread over 455 different scaffolds belonging to the seven chromosomes of the species (Veeckman, not published).

Genotypic data were recoded numerically to denote allelic doses [e.g., 0, 1, 2 for AA, AT, TT (A/T SNP)]. Imputation of missing genotype calls was performed independently for each group found by population structure analysis (see below). For each marker, a missing genotype call was replaced by the value of the most common allelic dose of its group. Minimal allele frequency (MAF) was set to 1% and markers with MAF below 1% were removed (Supplementary Table 3).

#### Correction for Heading Date in Spring

**FIGURE 1:**
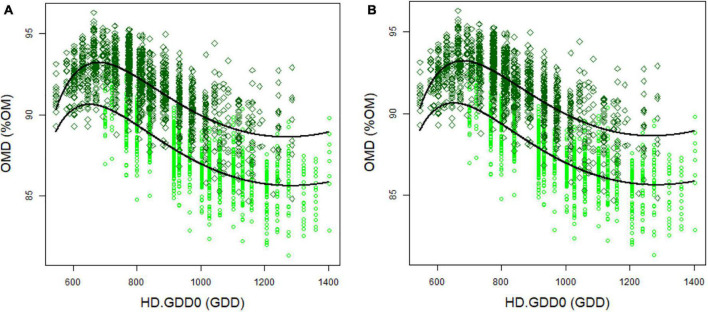
Organic matter digestibility (OMD) **(A)** and fibre digestibility (NDFD) **(B)** depending on heading date (HD.GDD0) in 2012 (light green circles) and 2013 (dark green diamonds). Black curves represent Eq. 6.

The harvest date in spring was set at the heading date of each individual genotype, as this is also an agronomic important time point at which farmers tend to harvest perennial ryegrass. However, this individual harvest date had an impact on OMD, NDFD, and NDF (Supplementary Table 4). In particular, OMD and NDFD decreased at later heading dates (Figure 1). To detect QTL not related to heading date in GWAS, we corrected each trait for heading date (HD). After optimisation, HD-corrected BLUP values were calculated with a harvest date correction using two models:

per season and per year:

(6) $$-Y_{il}=\mu +G_{i}+B_{l}+HD_{il}^{-2}+HD_{il}^{-1}+HD_{il}+e_{il}$$

per season over the two years:

(7) $$\begin{array}{c} -Y_{ijl}=\mu +G_{i}+T_{j}+GT_{ij}+B_{l}\left(T_{j}\right)+HD_{ijl}^{-2}+\\ HD_{ijl}^{-1}+HD_{ijl}+e_{ijl}, \end{array}$$

where *HD*_*il*_ = heading date of genotype *i* in block *l* per year,

*HD*_*ijl*_ = heading date of genotype *i* in block *l* in year *j.*

#### Population Structure

To determine population structure, we used fastSTRUCTURE (Raj et al., 2014) with default parameters for one to ten groups overall 580 genotypes. Three runs were performed to obtain a good prediction for the choice of the number of populations to retain.

Four genetic groups were identified, based on 252,406 SNPs and 5,074 indels, using marginal likelihood (Supplementary Figure 1). The first group, called “Temperate,” integrates all wild genotypes, except the ones from Spain and two from Italy, which belong to the second group named “Warm”. In addition, the “Temperate” group contains 16 genotypes from Barenbrug, 93 from DSV, 68 from ILVO, one from IBERS, nine from DLF, and two from Mommersteeg. The group “Warm” contains 33 genotypes from Barenbrug and one from DSV. The third group contains all genotypes derived from Aberavon, Aberdert, and Aberzest and is called “Aber” (Supplementary Table 1). It contains one genotype from Barenbrug, 27 from DSV, 41 from ILVO and 32 from IBERS. Most of the breeding genotypes and genotypes from commercial forage varieties are divided among these three groups. A fourth group, called “NZ,” contains 51 Barenbrug breeding genotypes from New Zealand. The principal component 1 (PC1) of the PCA from genotypic data (Supplementary Figure 2) indicated divergence between Aber, Temperate, and the two last groups (Warm and NZ). PC2 showed a divergence between four wild genotypes (two from Italy and two from Spain). PC3 showed a divergence between the Temperate group and the others.

#### Multi-Locus Mixed-Modelling Method

Multi-locus mixed-modelling (MLMM) was used for GWAS analysis (Segura et al., 2012). We used an Identity-by-State (IBS) matrix as kinship, computed using the formula from Rincent et al. (2012):


$$\mathrm{IBS}=\frac{GG^{\prime }+G_{2}G_{2}^{\prime }}{K},$$

where *G* is the matrix of genotypes (with individuals in rows and markers in columns) coded as 0, 0.5, and 1 for the homozygote, the heterozygote, and the alternative homozygote state, respectively; *K* is the total number of markers, and *G*_2_ = 1 - *G*, where 1 is a matrix of ones.

Multi-locus mixed-modelling was used for each trait with: (a) the matrix of genotype calls coded as 0, 1, and 2; (b) the BLUPs values; (c) the population structure; (d) the kinship matrix. The MLMM R function (Segura et al., 2012) was used. The best MLMM model with the lowest BIC value was retained for each trait. In addition to the QTLs identified in the best model, other QTLs were identified by using a Bonferroni threshold computed with the number of genes (503) regarding those markers on the same gene that had high linkage disequilibrium and the Bonferroni threshold is normally computed with the number of independent markers. The threshold for a global *p*-value of 5% was 0.05/503 = 9.94e10^–5^.

To evaluate the percentage of variance explained by the markers, first the most significant marker per gene was identified, and then, the log-likelihood of the global model with all identified QTLs and the log-likelihood of the model without the studied marker were computed with lmekin from the coxme R package using maximum likelihood (ML) (Therneau, 2020). Next, an estimation of the semi partial *R*^2^ of each studied marker was computed by a comparison of these models with the r.squaredLR function from the MuMIn R package (Bartoń, 2020).

For each trait, the effect of each QTL was estimated by the coefficient (slope) of the marker effect in the model.

To evaluate the effect of cell wall components QTLs on OMD and NDFD, two models were applied. For the spring cut, we evaluated the variance explained for OMD and NDFD by the QTLs identified for HC.NDF. In autumn, the same method was applied but used QTLs identified for ADL.NDF.

## Results

### Impact of the Season on Organic Matter Digestibility and Neutral Detergent Fiber Digestibility

The distribution of all traits per season and per year are given in Supplementary Figure 3 and summarised in Table 1. High variability among the 550 genotypes was observed for heading date, which was in this experiment the individual harvesting date per plant in spring.

**TABLE 1:** Descriptive data for organic matter digestibility (OMD), fibre content (NDF), fibre digestibility (NDFD), hemicellulose (HC), hemicellulose over NDF (HC.NDF), cellulose (C), cellulose over NDF (C.NDF), lignin (ADL), lignin over NDF (ADL.NDF), and heading date (HD) in growing degree days (GDD) from the potting date by a year and by season.

Season	Spring 2012					Spring 2013				
Trait	Mean	Min.	Max.	Var.	SD	Mean	Min.	Max.	Var.	SD
OMD (%OM)	87.42	81.34	92.90	3.65	1.91	91.67	84.69	96.30	3.66	1.91
NDF (%OM)	41.62	29.92	56.21	16.04	4.01	41.60	29.94	55.92	22.86	4.78
NDFD (%NDF)	69.86	63.10	78.84	7.77	2.79	80.00	71.00	89.94	13.41	3.66
HC (%OM)	23.27	18.06	30.76	3.81	1.95	23.98	16.76	37.79	11.17	3.34
HC.NDF (%NDF)	55.37	50.15	64.57	6.38	2.53	57.30	50.17	67.48	9.89	3.14
C (%OM)	17.35	11.19	25.54	5.39	2.32	16.23	10.99	24.75	4.67	2.16
C.NDF (%NDF)	42.08	32.48	47.45	6.64	2.58	39.27	27.34	46.85	12.66	3.56
ADL (%OM)	1.07	0.68	1.68	0.02	0.15	1.45	0.50	3.14	0.21	0.46
ADL.NDF (%NDF)	2.56	1.99	3.29	0.05	0.21	3.43	1.41	6.47	0.62	0.79
HD (GDD)	993	700	1402	26482	163	833	546	1286	23734	154

**Season**	**Autumn 2012**					**Autumn 2013**				
**Trait**	**Mean**	**Min.**	**Max.**	**Var.**	**SD**	**Mean**	**Min.**	**Max.**	**Var.**	**SD**

OMD (%OM)	78.12	66.46	87.04	12.06	3.47	84.49	67.98	93.53	12.57	3.55
NDF (%OM)	61.90	44.13	75.26	26.92	5.19	55.44	33.86	74.76	27.84	5.28
NDFD (%NDF)	64.83	53.07	73.50	10.28	3.21	72.25	53.04	83.27	18.38	4.29
HC (%OM)	33.63	25.38	41.20	9.02	3.00	29.95	18.99	39.16	9.55	3.09
HC.NDF (%NDF)	54.75	48.11	62.23	4.59	2.14	53.93	45.01	63.36	7.18	2.68
C (%OM)	25.69	18.37	33.89	5.79	2.41	23.74	15.85	32.65	6.60	2.57
C.NDF (%NDF)	41.52	32.24	49.18	6.80	2.61	42.65	32.41	51.78	8.41	2.90
ADL (%OM)	2.33	1.04	4.44	0.37	0.61	1.92	0.72	3.81	0.26	0.51
ADL.NDF (%NDF)	3.73	1.88	6.28	0.57	0.76	3.42	1.72	5.73	0.46	0.68

*Descriptive data are mean, minimum, (Min.), maximum (Max.), variance (Var.), and standard deviation (SD).*

The heritability of OMD and NDFD was very low or zero when both seasons were analysed together (Table 2, 0.13 and <0.01). Indeed, a strong genotype × season interaction (G:S) was observed. In particular, for OMD the G:S interaction was more than seven times higher than the genotypic variance. This strong interaction led us to analyse the data per season. The heritability of OMD and NDFD increased when analysed separately per season (Table 2), suggesting a different genetic control for these traits per season and the potential to breed separately for higher spring and/or autumn digestibility (OMD and NDFD).

**TABLE 2:** Heritabilities and variance components calculated from the mixed model Eq. 2 over seasons and Eq. 3 by season for OMD, NDF, NDFD, HC, HC.NDF, C, C.NDF, ADL, ADL.NDF, and HD in GDD from potting date.

Season	Spring and autumn					Spring				Autumn			
	Variance				*H* ^2^	Variance			*H* ^2^	Variance			*H* ^2^
Trait	G	G:Y	G:S	Res.		G	G:Y	Res.		G	G:Y	Res.	
OMD (%OM)	0.42	1.15	3.05	3.60	0.05	2.44	0.55	0.65	0.67	3.85	3.27	5.07	0.32
NDF (%OM)	4.39	3.89	6.24	8.43	0.19	7.99	6.99	4.93	0.40	11.36	5.53	8.92	0.44
NDFD (%NDF)	< 0.01	1.90	5.47	5.56	< 0.01	7.05	1.67	2.09	0.65	2.72	4.76	6.89	0.19
HC (%OM)	1.13	1.57	2.54	3.34	0.13	2.18	3.80	2.16	0.27	3.82	2.21	3.02	0.42
HC.NDF (%NDF)	1.60	0.65	2.73	2.20	0.22	5.51	1.51	1.60	0.64	2.86	0.84	2.19	0.49
C (%OM)	1.60	0.57	1.63	1.64	0.29	3.25	0.73	0.96	0.66	3.09	0.91	1.90	0.52
C.NDF (%NDF)	2.06	0.86	3.40	2.59	0.23	6.33	2.15	1.79	0.62	4.10	1.00	2.57	0.53
ADL (%OM)	< 0.01	0.04	0.09	0.09	< 0.01	0.02	0.06	0.04	0.17	0.14	0.07	0.10	0.45
ADL.NDF (%NDF)	< 0.01	0.07	0.18	0.19	< 0.01	0.06	0.14	0.14	0.18	0.23	0.12	0.17	0.44
HD (GDD)						21812	3319	1332	0.82				

*Are indicated: genotype effect (G), genotype × year interaction (G:Y), genotype × season interaction (G:S), and residuals (Res.).*

**FIGURE 2:**
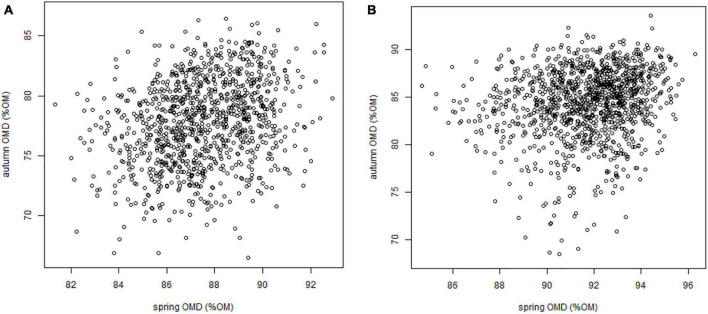
Organic matter digestibility of blades in spring against OMD of leaves in autumn in 2012 **(A)** and 2013 **(B)**.

The values of the digestibility traits OMD and NDFD were significantly different between the spring and autumn seasons. OMD was higher in spring than in autumn (8% OM difference), but the range of OMD was broader in autumn than in spring (11.6 and 23.1% OM, respectively). Similarly, NDFD was higher in spring than in autumn (6% NDF difference), but the range of NDFD was broader in autumn. Nevertheless, weak positive and significant correlations between seasons for OMD and NDFD were observed (Figure 2, Supplementary Table 5, and Supplementary Figure 4).

### Effect of Cell Wall Content and Cell Wall Digestibility on Spring and Autumn Digestibility

**FIGURE 3:**
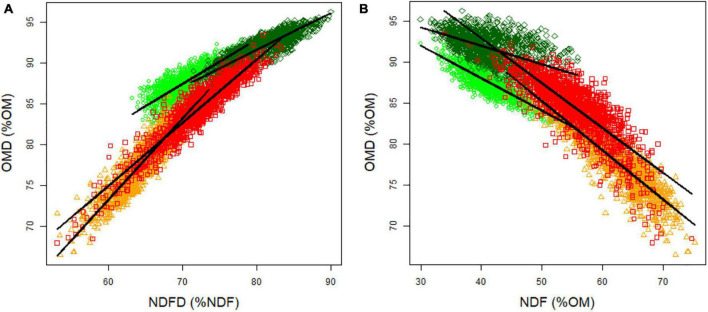
Relationship between OMD and **(A)** NDFD and **(B)** NDF. Light green circles indicate spring 2012 data, dark green diamonds indicate spring 2013 data, orange triangles indicate autumn 2012 data, and red squares indicate autumn 2013 data.

We observed a significant and strongly positive correlation between OMD and NDFD for each season and year [0.79 in spring 2012, 0.87 in spring 2013, 0.92 in autumn 2012, and 0.92 in autumn 2013 (*p* < 0.05); Figure 3A]. A significant and negative correlation was observed between OMD and NDF [-0.81 in spring 2012, -0.53 in spring 2013, -0.90 in autumn 2012 and -0.81 in autumn 2013 (*p* < 0.05); Figure 3B]. OMD could be explained by both the cell wall content (NDF) and the quality of the cell wall (NDFD) (Table 3). NDFD showed higher importance than NDF to explain OMD, except in spring 2012. Our data suggest that an increase of 1% OMD can be obtained by focusing on either a decrease of NDF or an increase of NDFD.

**TABLE 3:** Influence of fibre content and NDFD on OMD in %OM for each season by year (linear model): significance (*P*-val) and semi-partial *R*^2^ (sr^2^) for independent variables.

Season	Spring 2012		Spring 2013		Autumn 2012		Autumn 2013	
Trait	*P*-val	sr^2^	*P*-val	sr^2^	*P*-val	sr^2^	*P*-val	sr^2^
NDF (%OM)	***	0.37	***	0.24	***	0.15	***	0.12
NDFD (%NDF)	***	0.31	***	0.67	***	0.19	***	0.33
*R* ^2^	>0.99		>0.99		>0.99		>0.99	

*The coefficient of determination of the model (R^2^) is given.*

^ns^p > 0.05, *p < 0.05, **p < 0.01, ***p < 0.001.

**FIGURE 4:**
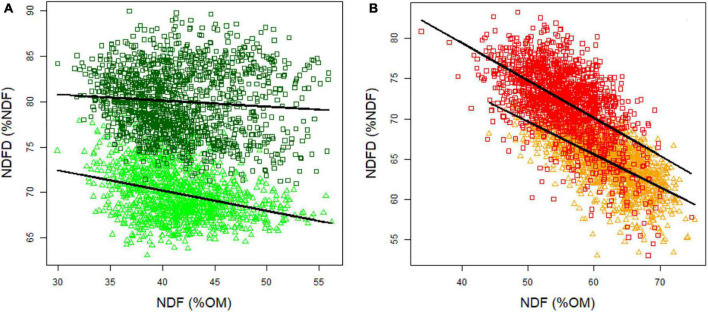
Relationship between NDF and NDFD in spring **(A)** and autumn **(B)**. Light green circles indicate spring 2012 data, dark green diamonds indicate spring 2013, orange triangles indicate autumn 2012 and red squares indicate autumn 2013 data.

Negative correlations between NDFD and NDF were observed for each season, but the coefficients were stronger in autumn (0.67 and -0.53 in 2012 and 2013, respectively) (Figure 4A) than in spring (-0.31 and -0.09 in 2012 and 2013, respectively) (Figure 4B). Despite this correlation, genotypes with similar NDF content were displayed in a wide range of NDFD values. Given the heritability of NDF and NDFD, breeding toward higher NDFD while maintaining the NDF content should be feasible in spring and holds promise for improving OMD in perennial ryegrass.

### The Effect of Cell Wall Composition on Neutral Detergent Fiber Digestibility

To understand better which aspects had determined the variation in NDFD in spring and autumn, we analysed the cell wall components. In spring, a large part of NDFD variability could be explained by HC.NDF itself (or C.NDF). A 1% increase of HC.NDF resulted in an increase of 0.81% of NDFD. In autumn, NDFD was mainly explained by ADL.NDF (Table 4). A 0.1% decrease of ADL.NDF resulted in an increase of 0.41% of NDFD. The studied population holds variation for HC.NDF in spring and ADL.NDF in autumn (Table 1). The heritabilities for these traits are high for HC.NDF (0.64 in spring), and moderate for ADL.NDF (0.44 in autumn) (Table 2).

**TABLE 4:** Influence of hemicellulose and lignin in fibres (HC.NDF and ADL.NDF) on NDFD in %NDF for each season by year (linear model): significance (*P*-val) and semi-partial *R*^2^ (sr^2^) for independent variables.

Season	Spring 2012		Spring 2013		Autumn 2012		Autumn 2013	
Trait	*P*-val	sr^2^	*P*-val	sr^2^	*P*-val	sr^2^	*P*-val	sr^2^
HC.NDF (%NDF)	***	0.39	***	0.46	ns	<0.01	***	0.04
ADL.NDF (%NDF)	***	<0.01	ns	<0.01	***	0.53	***	0.48
*R* ^2^	0.44		0.59		0.69		0.49	

*The coefficient of determination of the model (R^2^) is given.*

^ns^p > 0.05, *p < 0.05, **p < 0.01, ***p < 0.001.

### Genome-Wide Association Study

In spring, to identify QTLs different from the ones for heading date, and due to the correlation between traits and heading date, GWAS was performed on data corrected and uncorrected for heading date (Eqs 8, 9). All *p*-values, for all traits in spring and autumn, are presented in Supplementary Table 6 and the effect, the *p*-value, and the percentage of variance explained for all significant QTLs are presented in Supplementary Table 7. QTLs found could explain between 29 and 52% of the phenotypic variance (Table 5).

**TABLE 5:** Number of QTLs and genes identified by MLMM analysis and phenotypic variance explained by all QTLs identified (sr^2^) for each trait and season.

Season	Spring			Autumn		
Trait	QTLs	Genes	sr^2^	QTLs	Genes	sr^2^
OMD (%OM)	67	28	47%	57	21	40%
NDF (%OM)	109	23	52%	83	20	43%
NDFD (%NDF)	36	16	29%	52	19	35%
HC.NDF (%NDF)	60	18	39%	67	17	41%
C.NDF (%NDF)	54	16	38%	59	16	38%
ADL.NDF (%NDF)	96	27	49%	58	23	46%

Comparing all identified genes, we noticed that two genes (NAC-13, ABI3-02) were in common between HC.NDF, NDFD, and OMD in spring and six genes (RGA-03, ABI1-01, ARF-03, OBE1-02, KAN-06, and BIM2-01) were in common between ADL.NDF, NDFD, and OMD in autumn (Supplementary Table 8). In spring, 8.3% of OMD variance was explained by the three QTLs identified for both OMD and HC.NDF (NAC-13, SPL3-02, and ABI3-02). In autumn, 17.9% of OMD variance was explained by the seven QTLs identified for both OMD and ADL.NDF (RGA-03, ABI1-01, SPA-02, ARF-03, OBE1-02, MBD9-01, KAN-06, and BIM2-01). In spring, 6.3% of NDFD variance was explained by the three QTLs identified for both NDFD and HC.NDF (LUG-02, NAC-13, and ABI3-02). In autumn, 15.8% of NDFD variance was explained by the seven QTLs identified for both NDFD and ADL.NDF (RGA-03, ABI1-01, SPA-02, ARF-03, OBE1-02, KAN-06, and BIM2-01).

## Discussion

### Seasonal Differences in Organic Matter Digestibility and Neutral Detergent Fiber Digestibility Among Genotypes

We observed strong interactions between genotype and season for both OMD and NDFD, which leads to inversions of ranking between genotypes when autumn data versus spring data are considered. This observation indicates that selection should be realised in both spring and autumn simultaneously to improve the overall, annual digestibility. Interestingly, some genotypes showed high values of OMD and NDFD in both seasons, and are of particular relevance for breeding purposes.

The OMD was higher in spring than in autumn. This cannot be explained by the fact that only blades were analysed in spring compared to blades and sheaths combined in autumn since OMD of blades and “stems” (sheaths and flowering stems) was also higher in spring (van Parijs, 2016). This difference between seasons could be due to differences in environmental conditions (Buxton and Casler, 1993). Indeed, the autumn harvest is the result of the growth during summer (August and beginning of September), when temperatures were higher than in spring, which can lead to an increase in fibre content (Lee et al., 2017; Moyo and Nsahlai, 2021). The difference of irradiance at cutting can explain variability for soluble sugars concentration with higher values in spring than in autumn (Buxton and Casler, 1993), leading to the lower fibre content in spring.

We observed higher heritability in spring than in autumn for OMD and NDFD. This can be due to the high correlation between OMD and NDFD with heading date, with heading date being a highly heritable trait (*h*^2^ = 0.82) determined by a few genes with large effect (Wang and Forster, 2017).

The genetic variance of NDFD in spring was more than twice the value observed in autumn, while a higher genotype × year interaction was observed in autumn than in spring. This broad range of variation in spring NDFD suggests the potential for selection.

### Neutral Detergent Fiber Digestibility and Neutral Detergent Fiber Determine Organic Matter Digestibility in Both Spring and Autumn

The observed variation for OMD could be explained by both differences in the cell wall content (NDF) and of its digestibility (NDFD), as previously reported by Barrière et al. (2003) and Pembleton et al. (2013). Selection can be performed on both NDF and NDFD, but an appropriate selection approach should consider the fact that the correlation between NDFD and NDF was different in spring and autumn. In spring, the correlation between NDFD and NDF was weak, showing near independence between these two traits and allowing simultaneous selection for both NDFD and NDF. In contrast, NDFD and NDF were strongly negatively correlated in autumn, which would hamper simultaneous selection. A potential explanation of this difference in the strength of the correlation between these traits in different seasons lies in the difference in NDF and its variability between seasons. Biomass harvested in spring displays lower and less variable NDF values compared to biomass harvested in autumn. NDFD is on average higher in spring compared to autumn. We can assume a different cell wall biogenesis in summer when plants are resisting high temperatures and start preparing for winter survival. The autumn NDF content is high compared to spring when a high leaf growth rate can be observed associated with lower NDF content (Wingler and Hennessy, 2016).

Indeed, variability of NDFD can be partially explained by fibre composition, with the main contribution of different components in spring and autumn. In spring, an increase of NDFD was associated with an increase of HC (and a relative decrease of cellulose, given the negative correlation between both) but with almost no effect of lignin. In contrast, in autumn, an increase of NDFD was associated with a decrease of lignin concentration in the cell wall but with almost no effect of relative HC content. The link between cell wall lignin concentration and NDFD is normally observed in forage grasses (Mowat et al., 1969; Jung and Vogel, 1986), but not always (Lam et al., 2003). The absence of a link between cell wall lignin concentration and NDFD in spring is surprising, given that the same range for cell wall lignin concentration was observed in both spring and autumn. One explanation could be that the methodology used for cell wall characterisation is limiting because it is not precise enough. It is known that the ADL fraction determined with the Van Soest method detects the acid-insoluble lignin but does not allow quantifying the acid-soluble lignin (van Parijs et al., 2018). However, the effect of lignin on NDF digestibility depends not only on lignin concentration but also on cross-linking and hydrophobicity (Grabber et al., 2004). The acid-soluble phenolics are important in monocot species for cross-linking lignin with HC, while they are not detected by the van Soest method (van Parijs et al., 2018). Furthermore, the HC content in this study was estimated by substracting the ADF from the NDF content. This represents the detectable acid-soluble carbohydrates from the non-water-soluble biomass. Our HC content estimate may lack the HC fraction that is cross-linked with lignin. We can assume that in spring acid-soluble phenolics which are important in cross-linking lignin to HC play a role in the HC composition. Indeed, HC is a complex mixture of polysaccharides including xylans, β-glucans, xyloglucans, and mannans. arabinoxylans are predominant in HC in monocots and the high degree of substitution on the xylan backbone with arabinose relates to a low digestibility (Li et al., 2021). We observed an increase of NDFD associated with an increase of HC concentration, consistent with previous reports by Roussel et al. (2002). The effects observed in autumn were probably due to a major effect of the acid-insoluble lignin on cell wall digestibility, and not on the HC fraction. This may indicate that in autumn, the acid-insoluble lignin is better at determining the cell wall digestibility compared to the acid-soluble phenolics.

We observed a strong effect of heading date on all traits determined in plant materials harvested in spring. OMD decreased with a later heading date, which was associated with a decrease of NDFD, cell wall HC, and lignin concentrations. In other words, leaves harvested on late-flowering genotypes were less digestible than leaves harvested on early flowering genotypes. Since leaves were harvested at the heading stage, the decrease in digestibility could be explained by the age of leaves on later heading plants. Indeed, it has previously been reported that digestibility decreases with the increasing age of the organs (Buxton and Casler, 1993). It is therefore important to take into consideration the strong correlation between heading date and digestibility during breeding and selection. If this is not taken into account, breeding for highly digestible genotypes may indirectly lead to the selection of early heading material, which may or may not be desirable in the global breeding program.

### Genetic Control of Digestibility

The SNP dataset used in this study tagged genes from the pathways involved in plant cell wall biogenesis, plant architecture and development, and phytohormone biosynthesis, signalling, and response (Veeckman et al., 2018). GWAS identified multiple QTLs for each trait, spread across the genome, and explained together from 29.2 to 51.7% of phenotypic variance. Each marker, taken individually, explained a small part of phenotypic variance (from 1 to 7%). Markers identified by GWAS targeting to genes involved in different pathways. In general, the QTL effects were lower than the one observed in previous studies on *L. perenne*, as expected on mapping populations (Shinozuka et al., 2012), but also diversity panel (Pembleton et al., 2013). Several genes showing significant effects for the different quality traits considered in this study were also identified in other studies on digestibility and cell wall digestibility in forage grasses such perennial ryegrass and maize (Ralph et al., 2004; Pembleton et al., 2013). These include genes involved in lignin and cellulose biosynthesis (e.g., HCT3 and CAD in autumn OMD and C3H, ALDH and CCoAOMT in autumn NDFD; CAD2, COMT-01, ALDH, CES-02, XylT4, and PAL-03 in spring OMD and CES-02 and HCT in spring NDFD), which have previously been described to be involved in digestibility traits (reviewed by Halpin, 2019). Associations were also found for transcription factors such as WRKY-02, MYB-10, and NAC-13 with spring-related forage quality traits, and MYB-02 in autumn quality traits. These transcription factors have previously been described to be involved in cell wall biogenesis in grasses. For example, NAC transcription factors have been identified as master switches to grass secondary wall biogenesis by activating involved genes and transcription factors (Rao and Dixon, 2018). Furthermore, Myb transcription factors are considered as strong activators of cell wall biogenesis, though at a lower level than NAC (Nakano et al., 2015; Rao and Dixon, 2018). It has also been shown that members of the WRKY transcription factor family can repress lignification (Gallego-Giraldo et al., 2016; Rao and Dixon, 2018). A more in-depth analysis of these gene families in cell wall biosynthesis, including transcription factor families, may shed light on the regulation of secondary cell wall biogenesis in *L. perenne.*

No identical QTLs were identified across seasons, except one QTL for ADL.NDF, C.NDF, and OMD, and four QTLs for NDF. Within each season, we identified some QTLs common between digestibility traits and cell wall composition traits. In spring, HC.NDF had three QTLs in common with OMD and/or NDFD, whereas no common QTLs were identified in autumn. In autumn, ADL.NDF had seven QTLs in common with OMD and/or NDFD whereas no common QTL was identified in spring. These GWAS results confirm the importance of HC concentration for the digestibility of the spring cut, and lignin concentration in NDF for the digestibility of the autumn cut. QTLs only linked to NDFD or OMD could suggest there are other components, not considered in this study, which have a link with NDFD or OMD.

In conclusion, we found that NDF and NDFD are major components of OMD in spring and autumn, but we observed a high interaction between genotypes and seasons. In spring, NDFD was mainly explained by HC.NDF and C.NDF and their related QTLs. In autumn, NDFD was mainly explained by ADL.NDF and their related QTLs. This new knowledge suggests that breeding for OMD should be performed by selecting both NDF and NDFD in spring and autumn with an emphasis on cell wall HC content in spring and cell wall lignin content in autumn. In future work, the diversity of candidate genes identified in this study could be surveyed more in detail and other plant materials to identify interesting alleles useful for molecular-assisted breeding.

## Data Availability Statement

The original contributions presented in the study are included in the article/Supplementary Material, further inquiries can be directed to the corresponding author.

## Author Contributions

FP, HM, TR, and IR-R designed and performed the research. VC, PB, FP, TR, IR-R, and HM performed the experiments and analyzed the data. VC, PB, AE, and HM wrote the manuscript. All authors contributed to the article and approved the submitted version.

## Conflict of Interest

The authors declare that the research was conducted in the absence of any commercial or financial relationships that could be construed as a potential conflict of interest.

## Publisher’s Note

All claims expressed in this article are solely those of the authors and do not necessarily represent those of their affiliated organizations, or those of the publisher, the editors and the reviewers. Any product that may be evaluated in this article, or claim that may be made by its manufacturer, is not guaranteed or endorsed by the publisher.

## Abbreviations

ADF: acid detergent fibre
ADM: absolute dry matter
ADL: acid detergent lignin
ADL.NDF: cell wall ADL content
C: cellulose
C.NDF: cell wall cellulose content
CAD: carbamoyl-phosphate synthetase 2, aspartate transcarbamylase, and dihydroorotase
COMT: catechol-*O*-methyltransferase
DM: dry matter
DMD: dry matter digestibility
GWAS: Genome-Wide Association Study
HC: hemicellulose
HC.NDF: cell wall hemicellulose content
MLMM: multi-locus mixed-model
NDF: neutral detergent fibre (cell walls)
NDFD: neutral detergent fibre digestibility (cell wall digestibility)
NIRS: near-infrared spectroscopy
OM: organic matter
OMD: organic matter digestibility
QTL: quantitative trait loci
WSC: water-soluble carbohydrates.

## References

[B1] Akin D. E. Fales S. L. Rigsby L. L. Snook M. E. (1987). Temperature effects on leaf anatomy, phenolic acids, and tissue digestibility in tall fescue. *Agron. J.* 79 271–275. 10.2134/agronj1987.00021962007900020019x

[B2] Arojju S. K. Cao M. Zulfi Jahufer M. Z. Barrett B. A. Faville M. J. (2020). Genomic predictive ability for foliar nutritive traits in perennial ryegrass. *G3* 10 695–708. 10.1534/g3.119.400880 31792009 PMC7003077

[B3] Barrière Y. Guillet C. Goffner D. Pichon M. (2003). Genetic variation and breeding strategies for improved cell wall digestibility in annual forage crops. a review. *Anim. Res.* 52 193–228. 10.1051/animres:2003018

[B4] Bartoń K. (2020). *MuMIn: Multi**-Model Inference. R Package Version 1.43.17.* Available online at: https://CRAN.R-project.org/package=MuMIn (accessed July 27, 2021).

[B5] Bates D. Mächler M. Bolker B. Walker S. (2015). Fitting linear mixed-effects models using lme4. *J. Stat. Softw.* 67 1–48. 10.18637/jss.v067.i01

[B6] Beecher M. Baumont R. O’Donovan M. Boland T. M. Aufrère J. Fleming C. (2018). Effects of harvesting perennial ryegrass at different levels of herbage mass on voluntary intake and *in vivo* digestibility in sheep. *Grass Forage Sci.* 73 553–561. 10.1111/gfs.12319

[B7] Bonawitz N. D. Chapple C. (2010). The genetics of lignin biosynthesis: connecting genotype to phenotype. *Annu. Rev. Genet.* 44 337–363. 10.1146/annurev-genet-102209-163508 20809799

[B8] Bruinenberg M. H. Valk H. Korevaar H. Struik P. C. (2002). Factors affecting digestibility of temperate forages from seminatural grasslands: a review. *Grass Forage Sci.* 57 292–301. 10.1046/j.1365-2494.2002.00327.x

[B9] Buxton D. R. Casler M. D. (1993). “Environmental and genetic effects on cell wall composition and digestibility,” in *Forage Cell Wall Structure and Digestibility*, eds Jung H. G. Buxton D. R. Hatfield R. D. Ralph J. (Madison, WI: American Society of Agronomy), 685–714. 10.2134/1993.foragecellwall.c25

[B10] Carrère P. Pontes L. da S. Andueza D. Louault F. Rosseel D. (2010). Evolution de la valeur nutritive de graminées prairiales au cours de leur cycle de développement. *Fourrages* 201 27–35.

[B11] Casler M. D. Vogel K. P. (1999). Accomplishments and Impact from breeding for increased forage nutritional value. *Crop Sci.* 39 12–20. 10.2135/cropsci1999.0011183X003900010003x 34798789

[B12] Chen L. Auh C.-K. Dowling P. Bell J. Chen F. Hopkins A. (2003). Improved forage digestibility of tall fescue (Festuca arundinacea) by transgenic down-regulation of cinnamyl alcohol dehydrogenase. *Plant Biotechnol. J.* 1 437–449. 10.1046/j.1467-7652.2003.00040.x 17134402

[B13] Chen L. Auh C.-K. Dowling P. Bell J. Lehmann D. Wang Z.-Y. (2004). Transgenic down-regulation of caffeic acid O-methyltransferase (COMT) led to improved digestibility in tall fescue (Festuca arundinacea). *Funct. Plant Biol.* 31 235–245. 10.1071/fp03254 32688895

[B14] Cogan N. O. I. Smith K. F. Yamada T. Francki M. G. Vecchies A. C. Jones E. S. (2005). QTL analysis and comparative genomics of herbage quality traits in perennial ryegrass (Lolium perenne L.). *Theor Appl Genet* 110 364–380. 10.1007/s00122-004-1848-9 15558228

[B15] Durand J.-L. Gonzalez-Dugo V. Gastal F. (2010). How much do water deficits alter the nitrogen nutrition status of forage crops? *Nutr. Cycl. Agroecosyst.* 88 231–243. 10.1007/s10705-009-9330-3

[B16] Fick G. W. Wilkens P. W. Cherney J. H. (1994). “Modeling forage quality changes in the growing crop,” in *Forage Quality, Evaluation, and Utilization*, ed. Fahey G. C. (Madison, WI: American Society of Agronomy).

[B17] Gallagher J. A. Turner L. B. Cairns A. J. Farrell M. Lovatt J. A. Skøt K. (2015). Genetic differentiation in response to selection for water-soluble carbohydrate content in perennial ryegrass (Lolium perenne L.). *Bioenerg. Res.* 8 77–90. 10.1007/s12155-014-9491-z

[B18] Gallego-Giraldo L. Shadle G. Shen H. Barros-Rios J. Corrales S. F. Wang H. (2016). Combining enhanced biomass density with reduced lignin level for improved forage quality. *Plant Biotechnol. J.* 14 895–904. 10.1111/pbi.12439 26190611 PMC11388942

[B19] Goering H. K. Van Soest P. J. (1970). *Forage Fiber Analysis: Apparatus, Reagents, Procedures and Some Applications.* Washington, DC: U.S.D.A. Agricultural Research Service.

[B20] González L. A. Manteca X. Calsamiglia S. Schwartzkopf-Genswein K. S. Ferret A. (2012). Ruminal acidosis in feedlot cattle: interplay between feed ingredients, rumen function and feeding behavior (a review). *Anim. Feed Sci. Technol.* 172 66–79. 10.1016/j.anifeedsci.2011.12.009

[B21] Grabber J. H. (2005). How do lignin composition, structure, and cross-linking affect degradability? A review of cell wall model studies. *Crop Sci.* 45 820–831. 10.2135/cropsci2004.0191 34798789

[B22] Grabber J. H. Ralph J. Hatfield R. D. Quideau S. (1997). p-Hydroxyphenyl, guaiacyl, and syringyl lignins have similar inhibitory effects on wall degradability. *J. Agric. Food Chem.* 45 2530–2532. 10.1021/jf970029v

[B23] Grabber J. H. Ralph J. Lapierre C. Barrière Y. (2004). Genetic and molecular basis of grass cell-wall degradability. I. Lignin–cell wall matrix interactions. *C. R. Biol.* 327 455–465. 10.1016/j.crvi.2004.02.009 15255476

[B24] Groot J. C. J. Neuteboom J. H. Deinum B. (1999). Composition and digestibility during ageing of consecutive leaves on the main stem of Italian ryegrass plants, growing undisturbed or regrowing after cutting. *J. Sci. Food Agric.* 79 1691–1697. 10.1002/(SICI)1097-0010(199909)79:12<1691::AID-JSFA423<3.0.CO;2-L

[B25] Halpin C. (2019). Lignin engineering to improve saccharification and digestibility in grasses. *Curr. Opin. Biotechnol.* 56 223–229. 10.1016/j.copbio.2019.02.013 30909119

[B26] Herbe-book (2021). *Herbe-Book.* Available online at: https://www.herbe-book.org/ (Accessed January 26, 2021)

[B27] Hisano H. Nandakumar R. Wang Z.-Y. (2009). Genetic modification of lignin biosynthesis for improved biofuel production. *In Vitro Cell. Dev. Biol. Plant* 45 306–313. 10.1007/s11627-009-9219-5

[B28] Humphreys M. O. (1989). Water-soluble carbohydrates in perennial ryegrass breeding. III. relationships with herbage production, digestibility and crude protein content. *Grass Forage Sci.* 44 423–430. 10.1111/j.1365-2494.1989.tb01942.x

[B29] Jung H. G. Vogel K. P. (1986). Influence of lignin on digestibility of forage cell wall material. *J. Anim. Sci.* 62 1703–1712. 10.2527/jas1986.6261703x 3733564

[B30] Keys J. E. Van Soest P. J. Young E. P. (1969). Comparative study of the digestibility of forage cellulose and hemicellulose in ruminants and nonruminants1. *J. Anim. Sci.* 29 11–15. 10.2527/jas1969.29111x 5356690

[B31] Lam T. B.-T. Iiyama K. Stone B. A. (2003). Hot alkali-labile linkages in the walls of the forage grass *Phalaris aquatica* and *Lolium perenne* and their relation to *in vitro* wall digestibility. *Phytochemistry* 64 603–607. 10.1016/S0031-9422(03)00301-7 12943783

[B32] Lee M. A. Davis A. P. Chagunda M. G. G. Manning P. (2017). Forage quality declines with rising temperatures, with implications for livestock production and methane emissions. *Biogeosciences* 14 1403–1417. 10.5194/bg-14-1403-2017

[B33] Lemaire G. Salette J. Sigogne M. Terrasson J.-P. (1984). Relation entre dynamique de croissance et dynamique de prélèvement d’azote pour un peuplement de graminées fourragères. I. — Etude de l’effet du milieu. *Agronomie* 4 423–430. 10.1051/agro:19840503

[B34] Li Y.-L. Yu Y.-K. Zhu K.-M. Ding L.-N. Wang Z. Yang Y.-H. (2021). Down-regulation of MANNANASE7 gene in Brassica napus L. enhances silique dehiscence-resistance. *Plant Cell Rep.* 40 361–374. 10.1007/s00299-020-02638-5 33392730

[B35] Lopez-Malvar A. Malvar R. A. Butron A. Revilla P. Pereira-Crespo S. Santiago R. (2021). Genetic dissection for maize forage digestibility traits in a Multi-Parent Advanced Generation Intercross (MAGIC) population. *Agronomy* 11:104. 10.3390/agronomy11010104

[B36] McDonald P. Henderson A. R. (1964). Determination of water-soluble carbohydrates in grass. *J. Sci. Food Agric.* 15 395–398. 10.1002/jsfa.2740150609

[B37] Méchin V. Argillier O. Hébert Y. Guingo E. Moreau L. Charcosset A. (2001). Genetic analysis and QTL mapping of cell wall digestibility and lignification in silage maize. *Crop Sci.* 41 690–697. 10.2135/cropsci2001.413690x 34798789

[B38] Miller L. A. Moorby J. M. Davies D. R. Humphreys M. O. Scollan N. D. MacRae J. C. (2001). Increased concentration of water-soluble carbohydrate in perennial ryegrass (Lolium perenne L.): milk production from late-lactation dairy cows. *Grass Forage Sci.* 56 383–394. 10.1046/j.1365-2494.2001.00288.x

[B39] Mowat D. N. Kwain M. L. Winch J. E. (1969). Lignification and *in vitro* cell wall digestibility of plant parts. *Can. J. Plant Sci.* 49 499–504. 10.4141/cjps69-082

[B40] Moyo M. Nsahlai I. (2021). Consequences of increases in ambient temperature and effect of climate type on digestibility of forages by ruminants: a meta-analysis in relation to global warming. *Animals* 11:172. 10.3390/ani11010172 33450884 PMC7828355

[B41] Nakano Y. Yamaguchi M. Endo H. Rejab N. A. Ohtani M. (2015). NAC-MYB-based transcriptional regulation of secondary cell wall biosynthesis in land plants. *Front. Plant Sci.* 6:288. 10.3389/fpls.2015.00288 25999964 PMC4419676

[B42] Newman Y. C. Lambert B. Muir J. P. (2006). *Defining Forage Quality.* Texas Cooperative Extension, The Texas A&M University System.

[B43] O’Grady L. Doherty M. L. Mulligan F. J. (2008). Subacute ruminal acidosis (SARA) in grazing Irish dairy cows. *Vet. J.* 176 44–49. 10.1016/j.tvjl.2007.12.017 18328751

[B44] Oba M. Allen M. S. (1999). Evaluation of the importance of the digestibility of neutral detergent fiber from forage: effects on dry matter intake and milk yield of dairy cows. *J. Dairy Sci.* 82 589–596. 10.3168/jds.S0022-0302(99)75271-9 10194678

[B45] Parsons A. J. Edwards G. R. Newton P. C. D. Chapman D. F. Caradus J. R. Rasmussen S. (2011). Past lessons and future prospects: plant breeding for yield and persistence in cool-temperate pastures: plant breeding for yield and persistence. *Grass Forage Sci.* 66 153–172. 10.1111/j.1365-2494.2011.00785.x

[B46] Pembleton L. W. Wang J. Cogan N. O. I. Pryce J. E. Ye G. Bandaranayake C. K. (2013). Candidate gene-based association genetics analysis of herbage quality traits in perennial ryegrass (Lolium perenne L.). *Crop Pasture Sci.* 64 244–253. 10.1071/CP12392

[B47] Pontes L. S. Carrère P. Andueza D. Louault F. Soussana J. F. (2007). Seasonal productivity and nutritive value of temperate grasses found in semi-natural pastures in Europe: responses to cutting frequency and N supply. *Grass Forage Sci.* 62 485–496. 10.1111/j.1365-2494.2007.00604.x

[B48] R Core Team (2020). *R: A Language and Environment for Statistical Computing.* Vienna: R Foundation for Statistical Computing.

[B49] Raj A. Stephens M. Pritchard J. K. (2014). fastSTRUCTURE: variational inference of population structure in large SNP data sets. *Genetics* 197 573–589. 10.1534/genetics.114.164350 24700103 PMC4063916

[B50] Ralph J. Guillaumie S. Grabber J. H. Lapierre C. Barrière Y. (2004). Genetic and molecular basis of grass cell-wall biosynthesis and degradability. III. towards a forage grass ideotype. *C. R. Biol.* 327 467–479. 10.1016/j.crvi.2004.03.004 15255477

[B51] Rao X. Dixon R. A. (2018). Current Models for transcriptional regulation of secondary cell wall biosynthesis in grasses. *Front. Plant Sci.* 9:399. 10.3389/fpls.2018.00399 29670638 PMC5893761

[B52] Riboulet C. Fabre F. Dénoue D. Martinant J. P. Lefevre B. Barrière Y. (2008). QTL mapping and candidate gene research for lignin content and cell wall digestibility in a top-cross of a flint maize recombinant inbred line progeny harvested at silage stage. *Maydica* 53 1–9.

[B53] Rincent R. Laloë D. Nicolas S. Altmann T. Brunel D. Revilla P. (2012). Maximizing the reliability of genomic selection by optimizing the calibration set of reference individuals: comparison of methods in two diverse groups of maize inbreds (Zea mays L.). *Genetics* 192 715–728. 10.1534/genetics.112.141473 22865733 PMC3454892

[B54] Roussel V. Gibelin C. Fontaine A. S. Barrière Y. Y. (2002). Genetic analysis in recombinant inbred lines of early dent forage maize. II-QTL mapping for cell wall constituents and cell wall digestibility from per se value and top cross experiments. *Maydica* 47 9–20.

[B55] Sampoux J.-P. Baudouin P. Bayle B. Béguier V. Bourdon P. Chosson J.-F. (2011). Breeding perennial grasses for forage usage: an experimental assessment of trait changes in diploid perennial ryegrass (Lolium perenne L.) cultivars released in the last four decades. *Field Crops Res.* 123 117–129. 10.1016/j.fcr.2011.05.007

[B56] Segura V. Vilhjálmsson B. J. Platt A. Korte A. Seren Ü Long Q. (2012). An efficient multi-locus mixed-model approach for genome-wide association studies in structured populations. *Nat. Genet.* 44 825–830. 10.1038/ng.2314 22706313 PMC3386481

[B57] Semae (2020). *Statistique Annuelle et Séries Chronologiques - Semences de Plantes Fourragères et Gazons - Campagne 2018/2019.* Available online at: https://www.gnis.fr/etudes-donnees-statistiques-semences/ (Accessed January 19, 2021)

[B58] Shinozuka H. Cogan N. O. Spangenberg G. C. Forster J. W. (2012). Quantitative Trait Locus (QTL) meta-analysis and comparative genomics for candidate gene prediction in perennial ryegrass (Lolium perenne L.). *BMC Genet.* 13:101. 10.1186/1471-2156-13-101 23137269 PMC3532372

[B59] Smirnova O. G. Kochetov A. V. (2016). Plant cell wall and the mechanisms of resistance to pathogens. *Vavilov J. Genet. Breed.* 19 715–723. 10.18699/VJ15.109

[B60] Struik P. C. (1983). *Physiology of Forage Maize (Zea mays L.) in Relation to its Production and Quality.* Available online at: https://library.wur.nl/WebQuery/wurpubs/fulltext/205777 (Accessed February 11, 2020).

[B61] Terry R. A. Tilley J. M. A. (1964). The digestibility of the leaves and stems of perennial ryegrass, cocksfoot, timothy, tall fescue, lucerne and sainfoin, as measured by an *in vitro* procedure. *Grass Forage Sci.* 19 363–372. 10.1111/j.1365-2494.1964.tb01188.x

[B62] Therneau T. M. (2020). *Coxme: Mixed Effects Cox Models. R Package Version 2.2-16.* Available online at: https://CRAN.R-project.org/package=coxme (accessed July 27, 2021).

[B63] Thomas C. Daley S. R. Aston K. Hughes P. M. (1981). Milk production from silage 2. the influence of the digestibility of silage made from the primary growth of perennial ryegrass. *Anim. Prod.* 33 7–13. 10.1017/S0003356100025137

[B64] Turner L. B. Cairns A. J. Armstead I. P. Ashton J. Skøt K. Whittaker D. (2006). Dissecting the regulation of fructan metabolism in perennial ryegrass (Lolium perenne) with quantitative trait locus mapping. *New Phytol.* 169 45–58. 10.1111/j.1469-8137.2005.01575.x 16390418

[B65] Ullmann I. Herrmann A. Hasler M. Taube F. (2017). Influence of the critical phase of stem elongation on yield and forage quality of perennial ryegrass genotypes in the first reproductive growth. *Field Crops Res.* 205 23–33. 10.1016/j.fcr.2017.02.003

[B66] van Parijs F. (2016). *Cell Wall Digestibility of Perennial Ryegrass?: An Association Mapping Approach.* Available online at: http://hdl.handle.net/1854/LU-7899090 (Accessed February 4, 2019)

[B67] van Parijs F. R. D. Waes C. V. Vandecasteele B. Haesaert G. Roldán-Ruiz I. Muylle H. (2018). The optimal lignin quantification method to breed for an improved cell wall digestibility in perennial ryegrass. *Grass Forage Sci.* 73 101–111. 10.1111/gfs.12293

[B68] Van Soest P. J. (1967). Development of a comprehensive system of feed analyses and its application to forages. *J. Anim. Sci.* 26 119–128. 10.2527/jas1967.261119x 32704858

[B69] Van Soest P. J. Robertson J. B. Lewis B. A. (1991). Methods for dietary fiber, neutral detergent fiber, and nonstarch polysaccharides in relation to animal nutrition. *J. Dairy Sci.* 74 3583–3597. 10.3168/jds.S0022-0302(91)78551-2 1660498

[B70] Veeckman E. Van Glabeke S. Haegeman A. Muylle H. van Parijs F. R. D. Byrne S. L. (2018). Overcoming challenges in variant calling: exploring sequence diversity in candidate genes for plant development in perennial ryegrass (*Lolium perenne*). *DNA Res.* 26 1–12. 10.1093/dnares/dsy033 30325414 PMC6379033

[B71] Vetharaniam I. Kelly W. J. Attwood G. T. Harris P. J. (2014). A 3-D model of a perennial ryegrass primary cell wall and its enzymatic degradation. *Computation* 2 23–46. 10.3390/computation2020023

[B72] Wang J. Forster J. W. (2017). Flowering time regulation in perennial ryegrass. *Euphytica* 213:106. 10.1007/s10681-017-1896-2

[B73] Wilkins P. W. (1997). Useful variation in *in vitro* digestibility within perennial ryegrass. *Euphytica* 93 249–255. 10.1023/A:1002910707241

[B74] Wilkins P. W. Humphreys M. O. (2003). Progress in breeding perennial forage grasses for temperate agriculture. *J. Agric. Sci.* 140 129–150. 10.1017/S0021859603003058

[B75] Wilman D. Altimimi M. A. K. (1982). The digestibility and chemical composition of plant parts in Italian and perennial ryegrass during primary growth. *J. Sci. Food Agric.* 33 595–602. 10.1002/jsfa.2740330702

[B76] Wingler A. Hennessy D. (2016). Limitation of grassland productivity by low temperature and seasonality of growth. *Front. Plant Sci.* 7:1130. 10.3389/fpls.2016.01130 27512406 PMC4962554

[B77] Wong D. (2005). *World Forage, Turf and Legume Seed Markets. Alberta Agriculture, Food and Rural Development.* Available online at: https://www1.agric.gov.ab.ca/%24department/newslett.nsf/pdf/fsu6885/%24file/worldforage.pdf (Accessed January 19, 2021).

[B78] Woodman H. E. Stewart J. (1932). The mechanism of cellulose digestion in the ruminant organism. III. the action of cellulose-splitting bacteria on the fibre of certain typical feeding stuffs. *J. Agric. Sci.* 22:527. 10.1017/S0021859600054071

