# Supplementary figures and images for: Seasonal Differences in Structural and Genetic Control of Digestibility in Perennial Ryegrass
Source: Front Plant Sci. 2022 Jan 4;12:801145. doi: 10.3389/fpls.2021.801145 (PMC8765707; doi:10.3389/fpls.2021.801145)

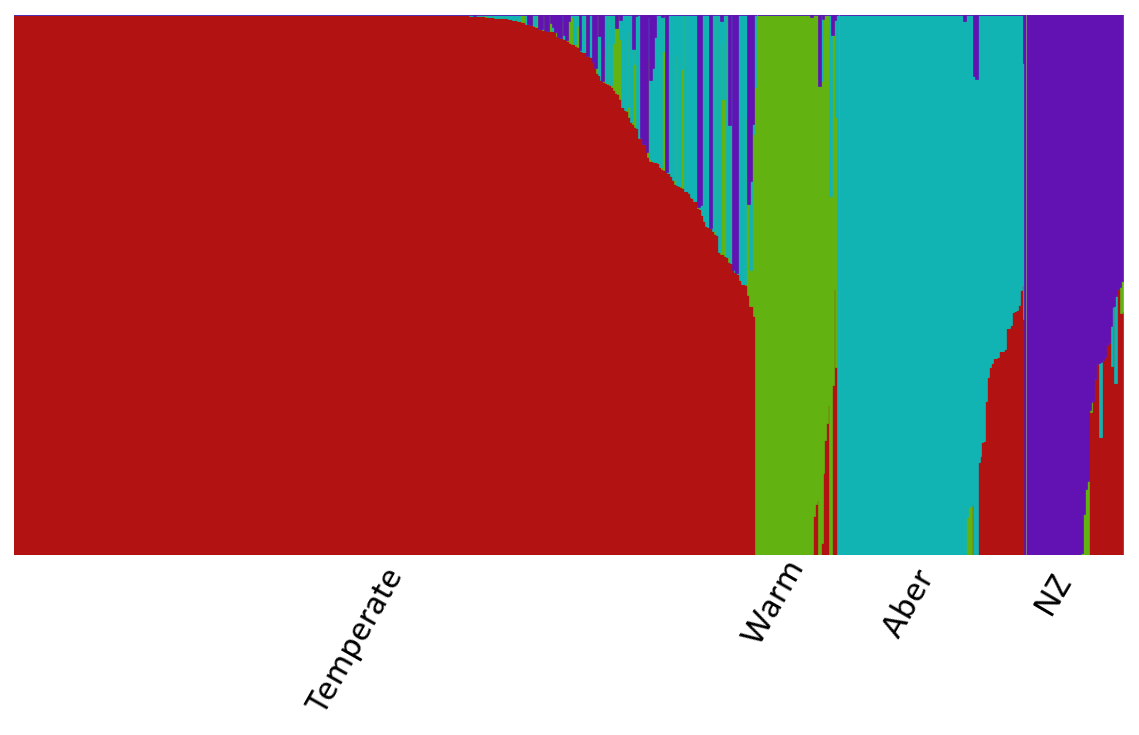

Supplement: Supplementary file 1 [file Image_1.png]

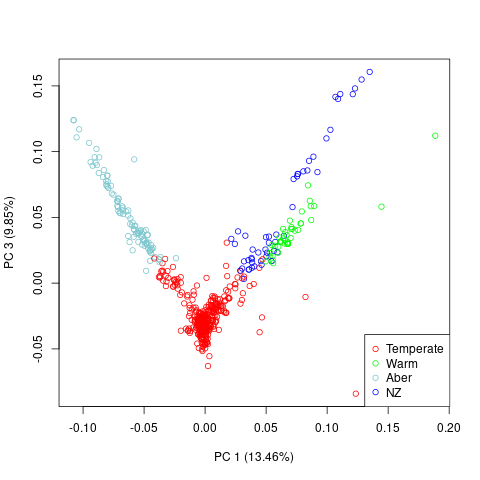

Supplement: Supplementary file 2 [file Image_2.png]

2012

2013

OMD (%OM)

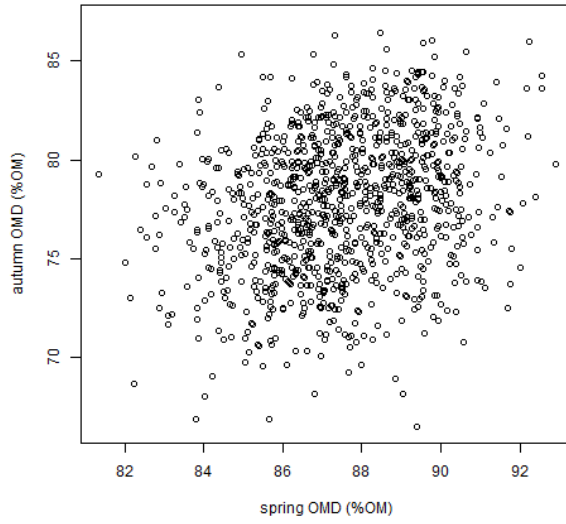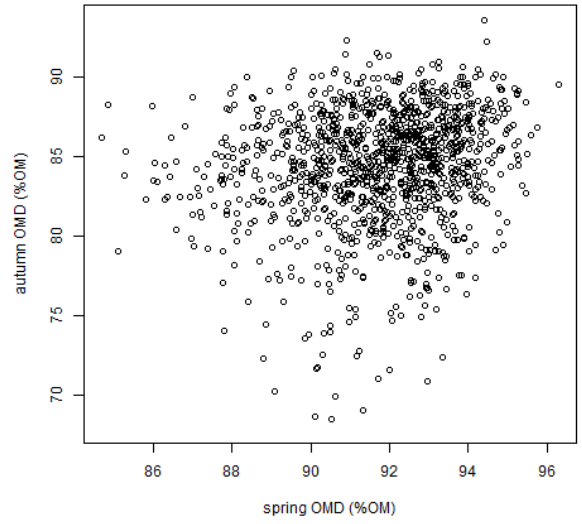

NDF (%OM)

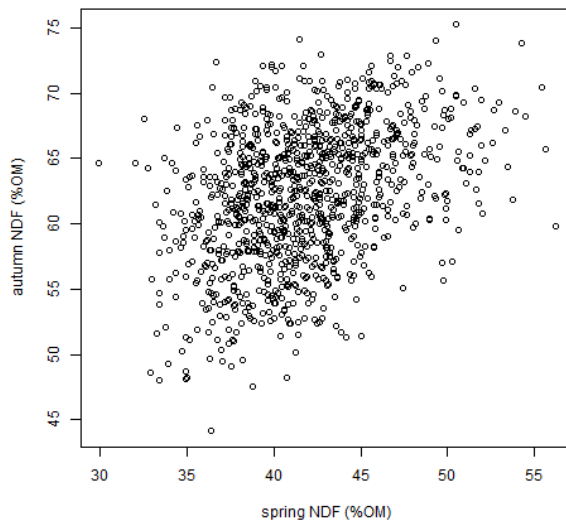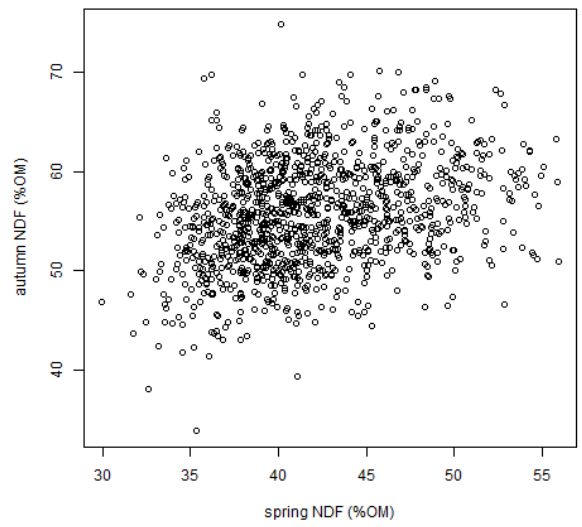

NDFD (%NDF)

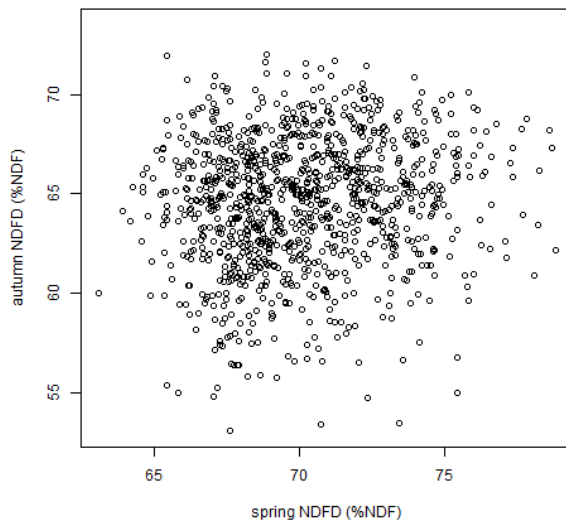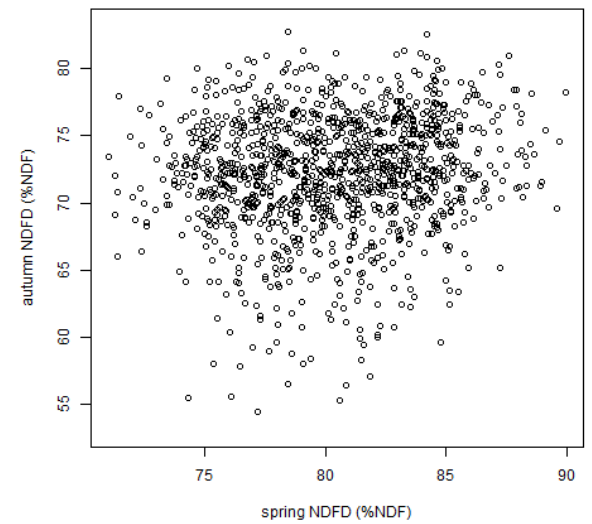

2012

2013

HC (%OM)

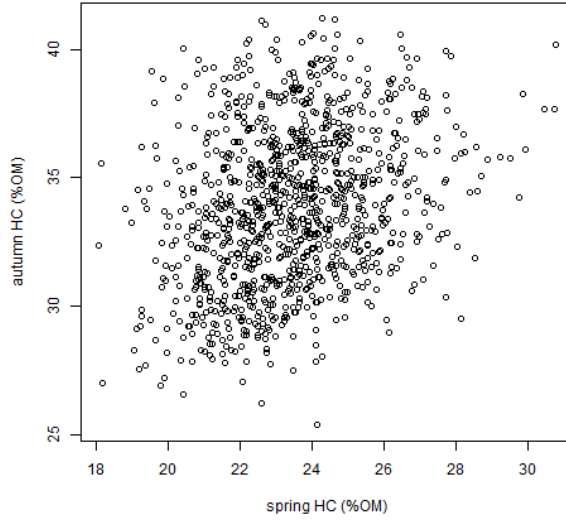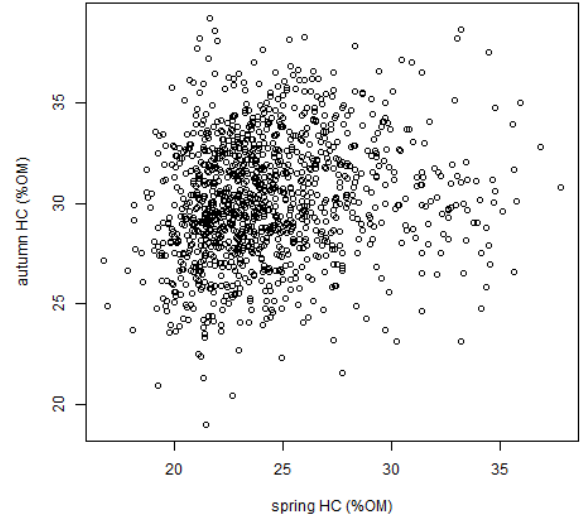

HC.NDF (%NDF)

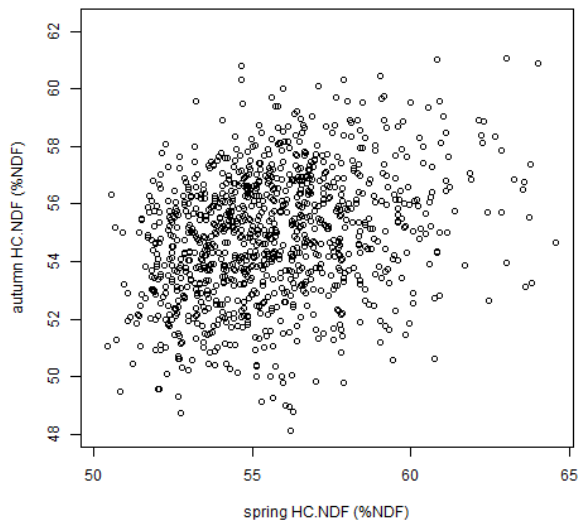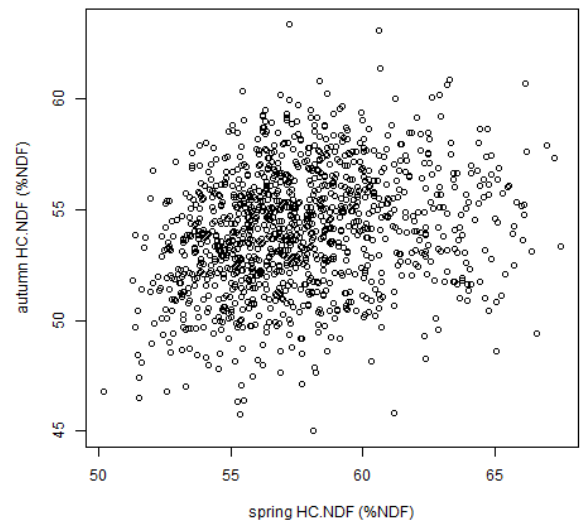

C (%OM)

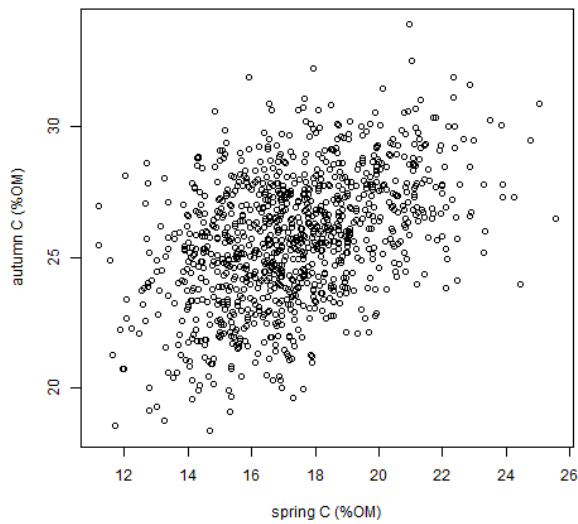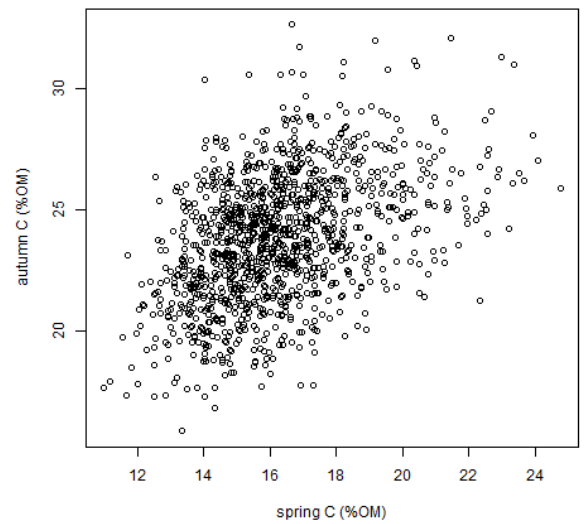

2012

2013

C.NDF (%NDF)

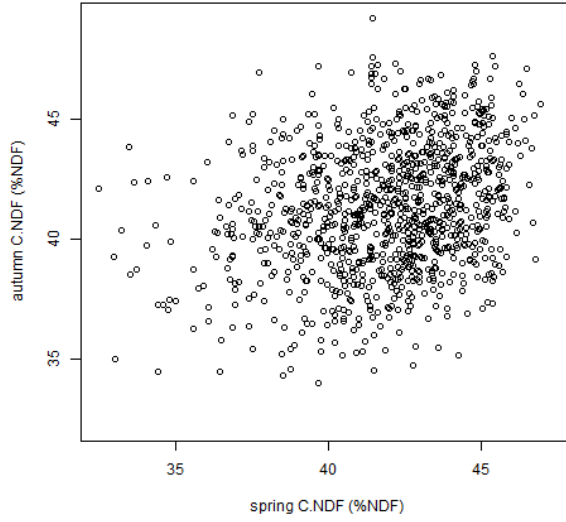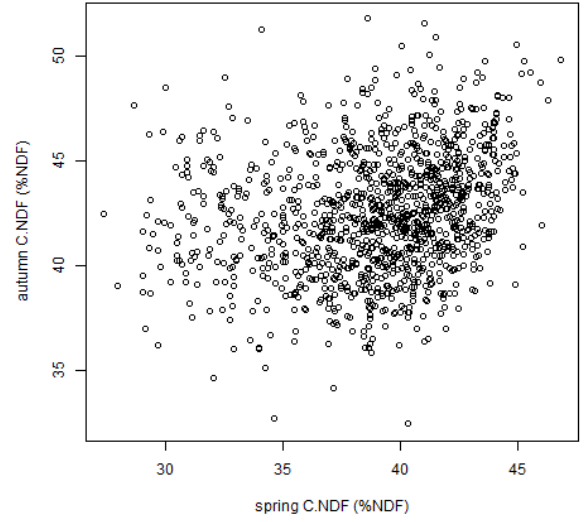

ADL (%OM)

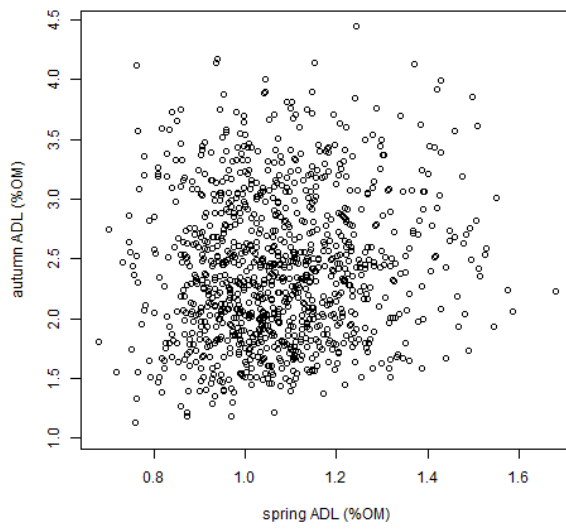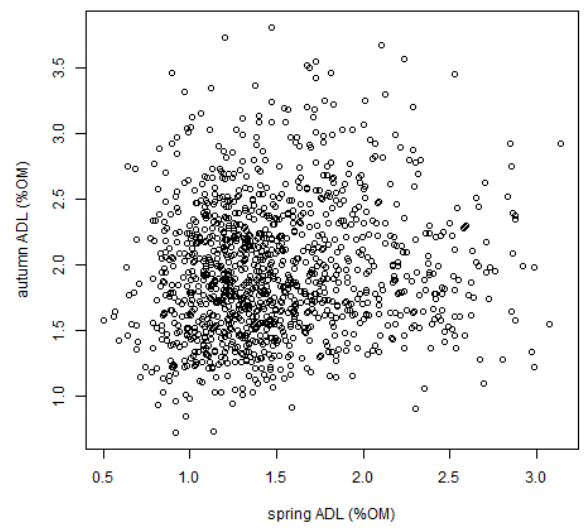

ADL.NDF (%NDF)

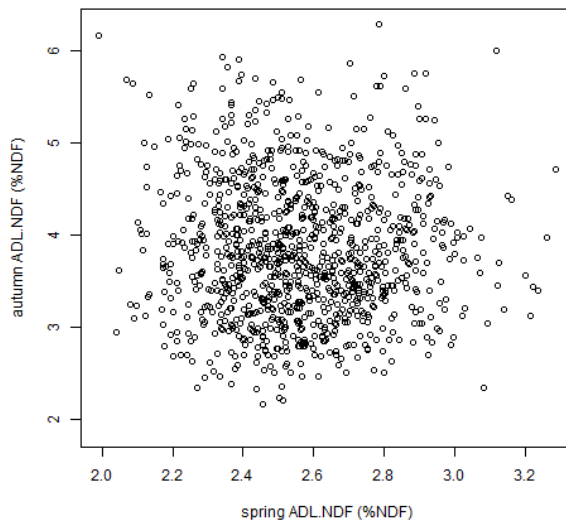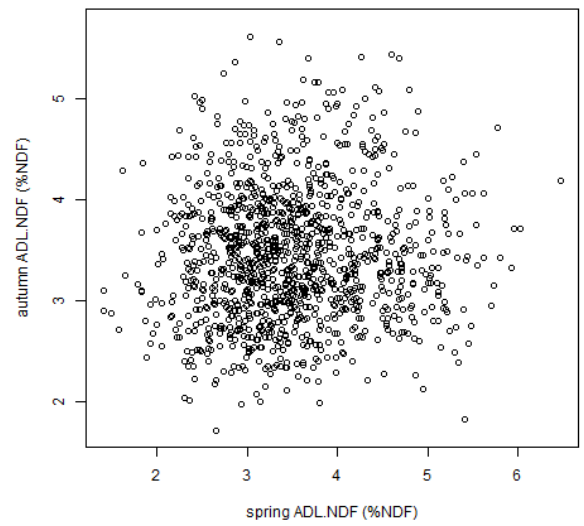

Supplement: Supplementary file 4 [file Image_4.pdf]
