# Supplementary material for: Seasonal Differences in Structural and Genetic Control of Digestibility in Perennial Ryegrass
Source: Front Plant Sci. 2022 Jan 4;12:801145. doi: 10.3389/fpls.2021.801145 (PMC8765707; doi:10.3389/fpls.2021.801145)
Supplement: Supplementary file 3 [file Image_3.pdf]

2012 2013

Spring
OMD (%OM)

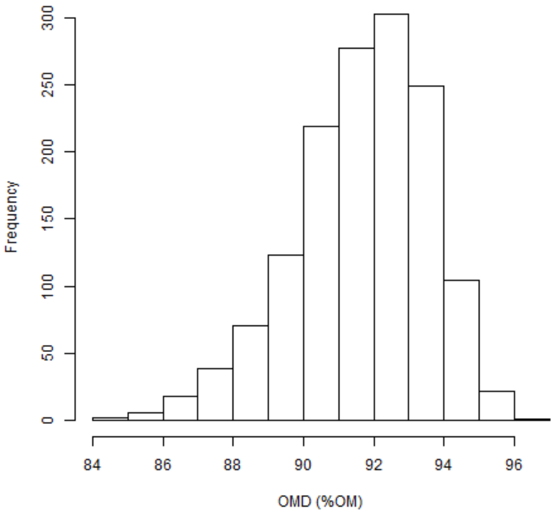

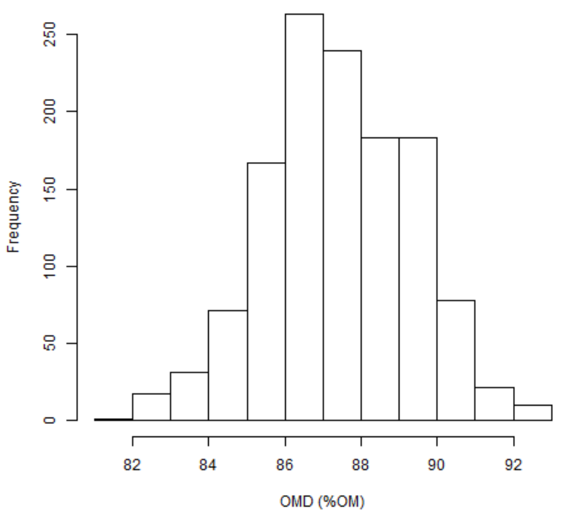

Spring
NDF (%OM)

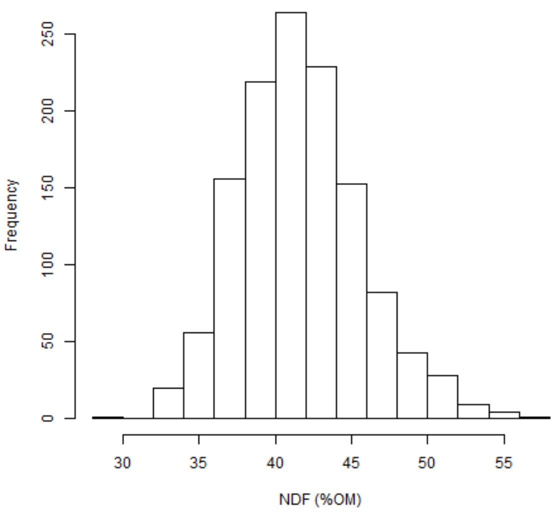

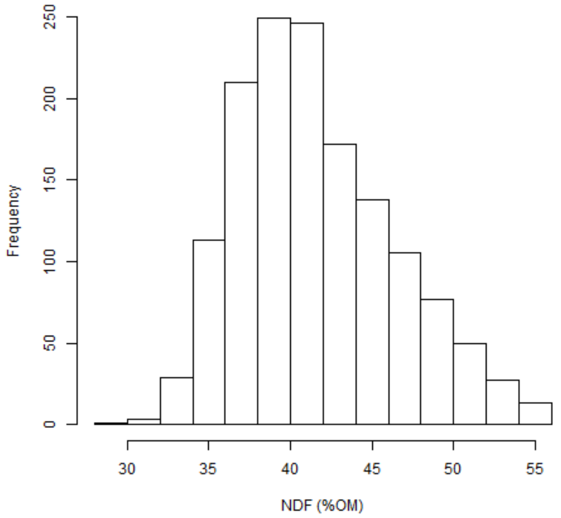

Spring
NDFD (%NDF)

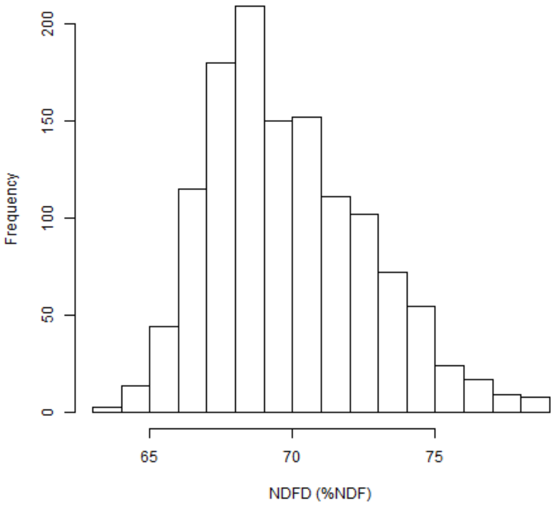

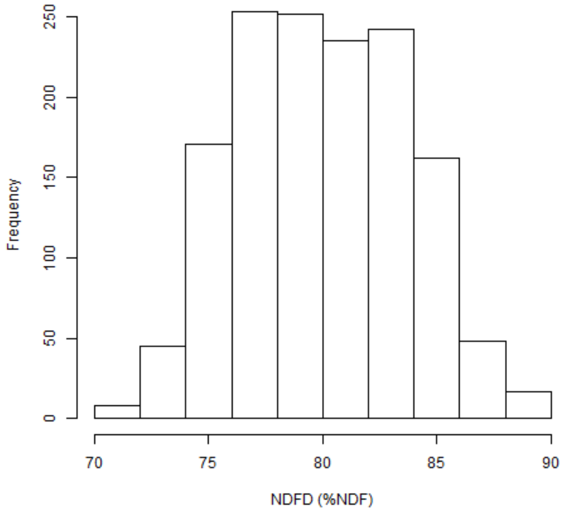

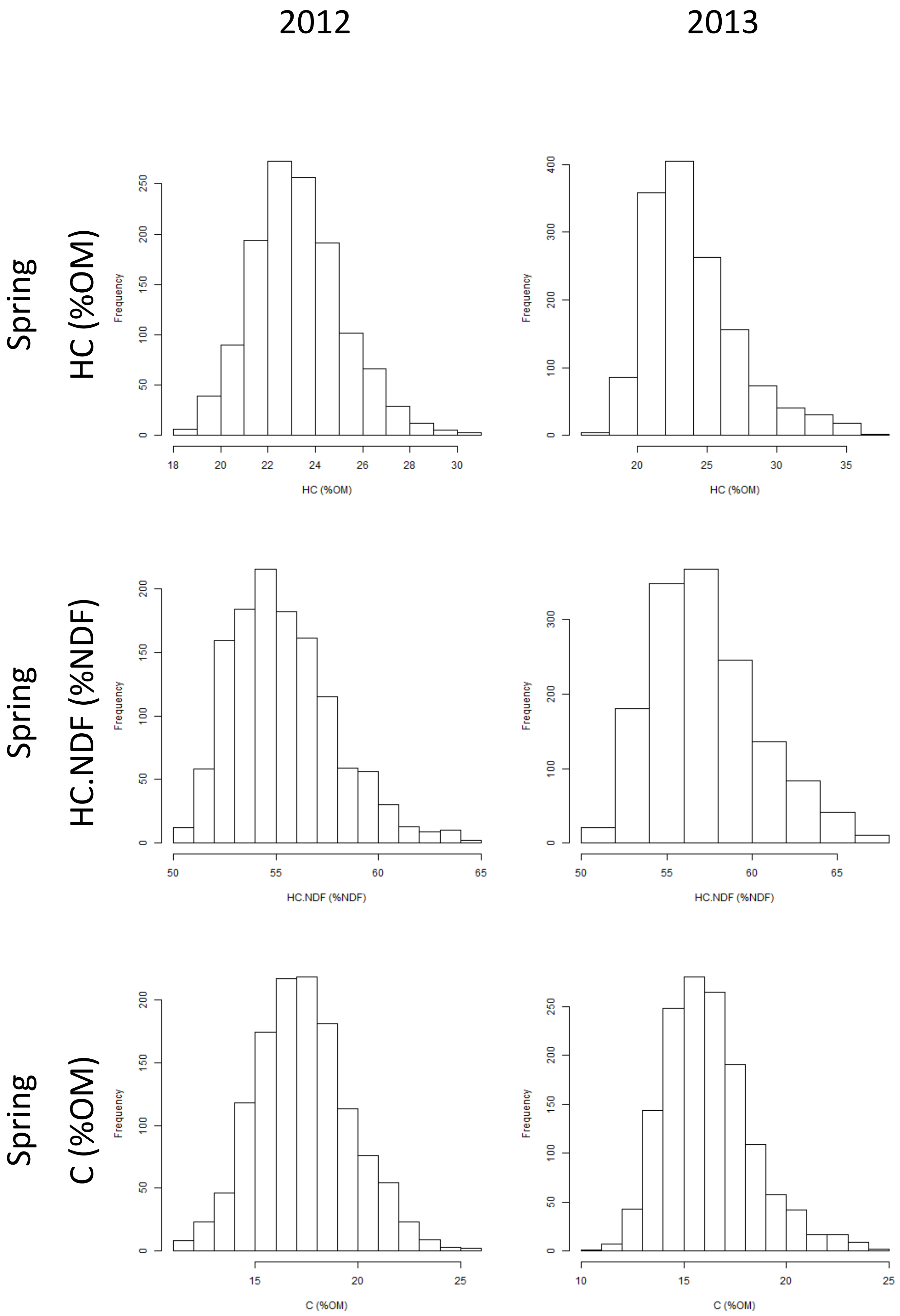
2012
2013
Spring
HC (%OM)
Frequency
HC (%OM)
Frequency
HC (%OM)
Spring
HC.NDF (%NDF)
Frequency
HC.NDF (%NDF)
Frequency
HC.NDF (%NDF)
Spring
C (%OM)
Frequency
C (%OM)
Frequency
C (%OM)

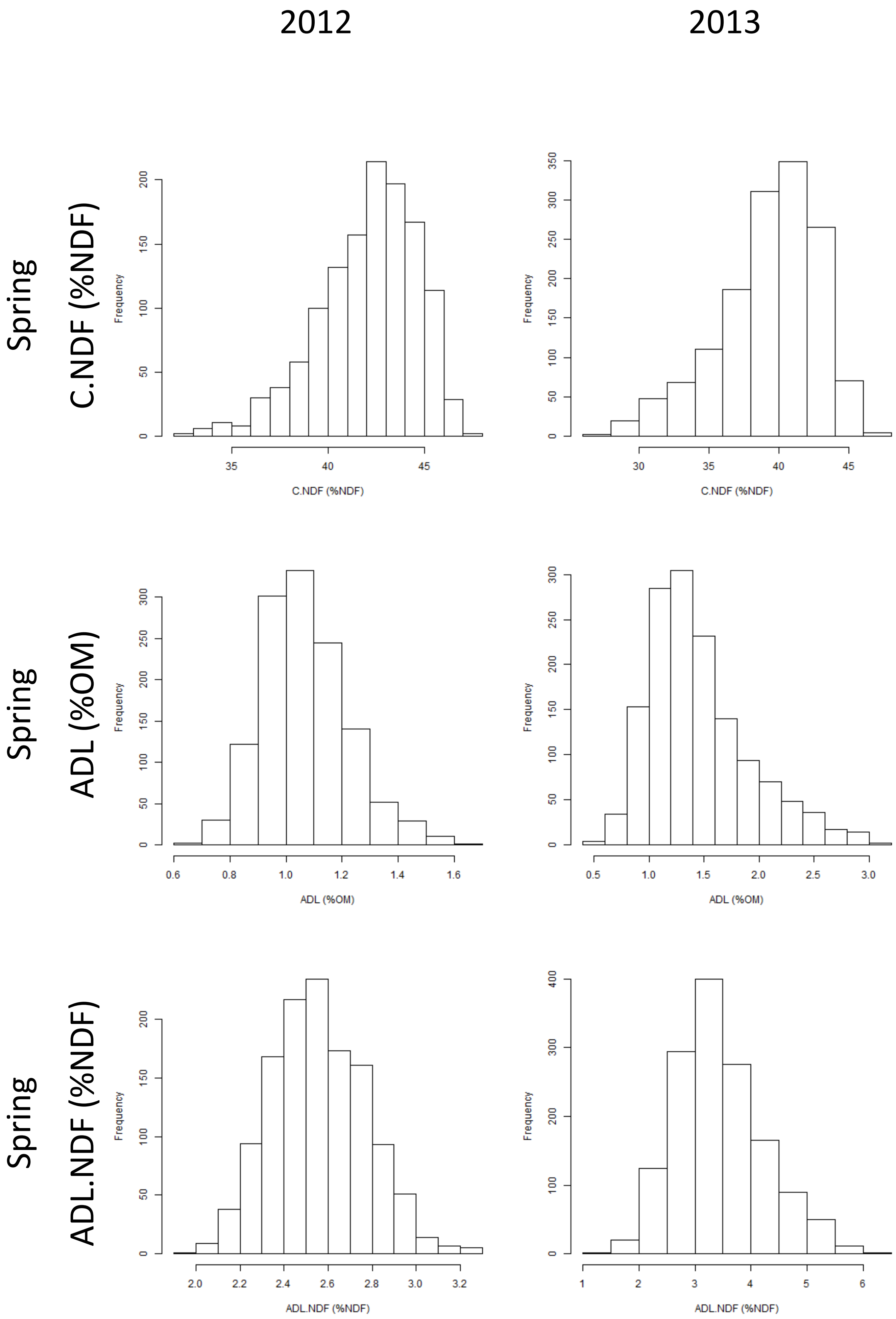
2012
2013
Spring
C.NDF (%NDF)
Spring
ADL (%OM)
Spring
ADL.NDF (%NDF)
Frequency
C.NDF (%NDF)
ADL (%OM)
ADL.NDF (%NDF)

2012 2013

Spring HD (GDD)

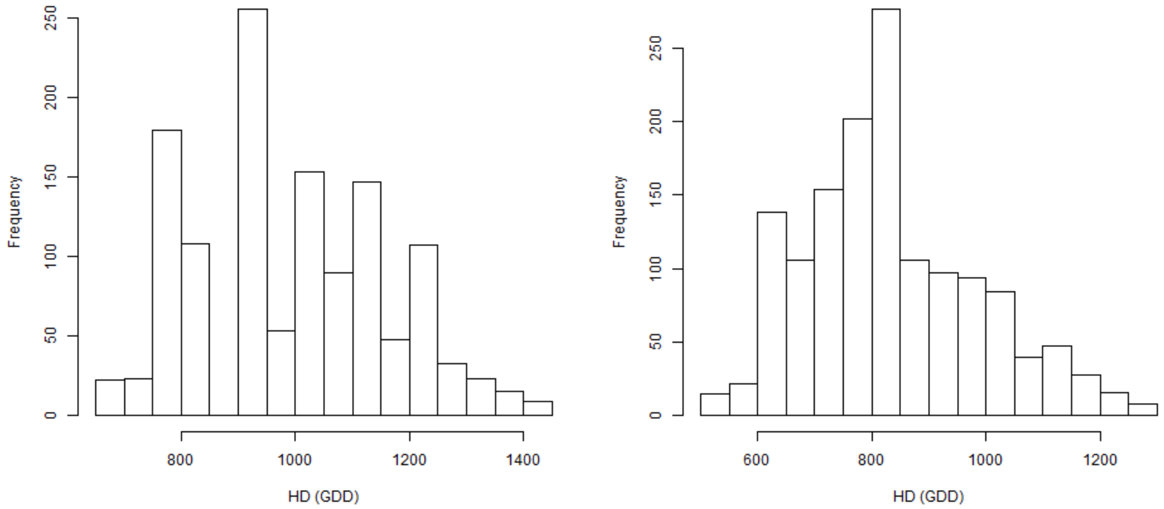

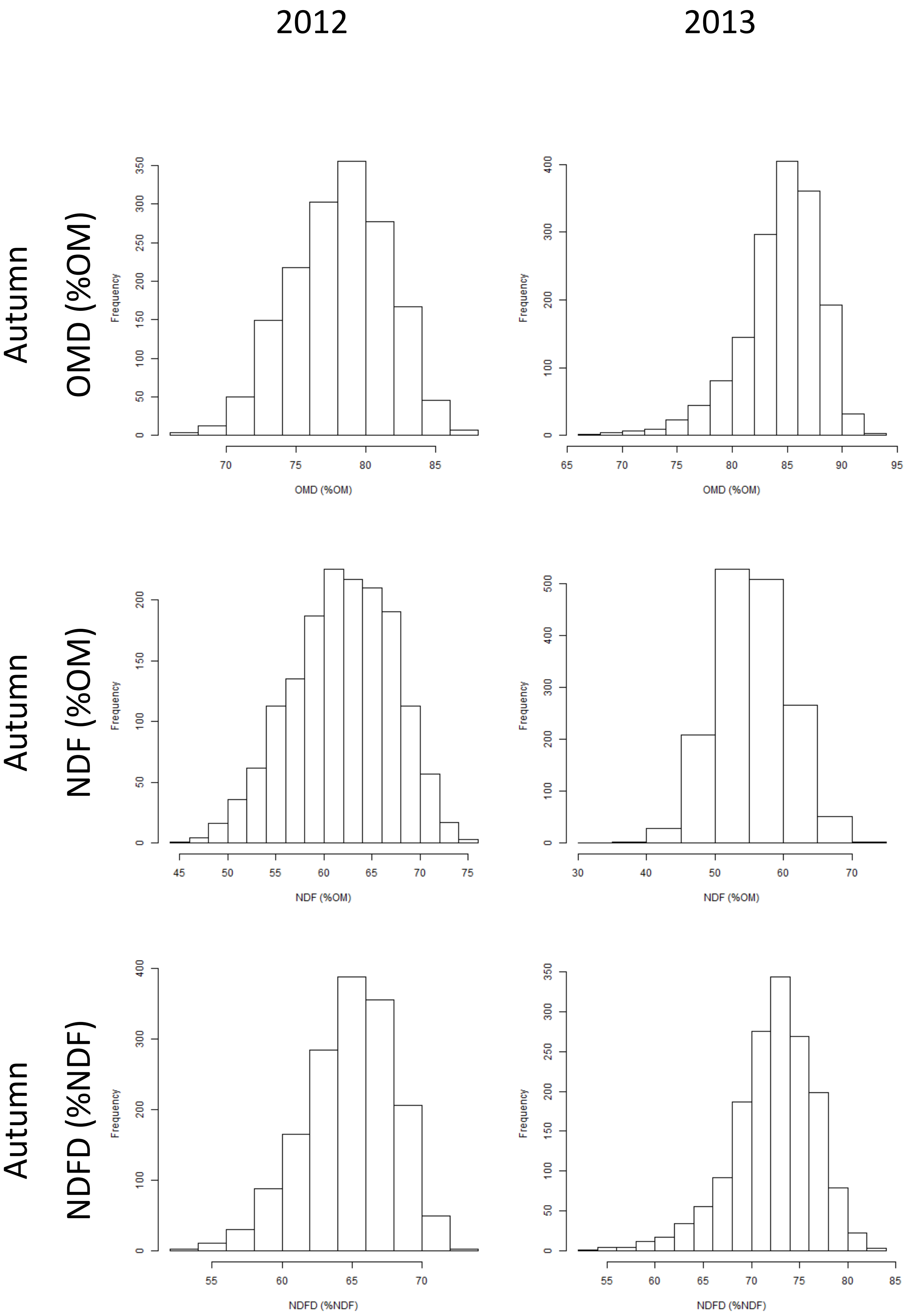
2012
2013
Autumn
OMD (%OM)
Autumn
NDF (%OM)
Autumn
NDFD (%NDF)
Frequency
OMD (%OM)
NDF (%OM)
NDFD (%NDF)

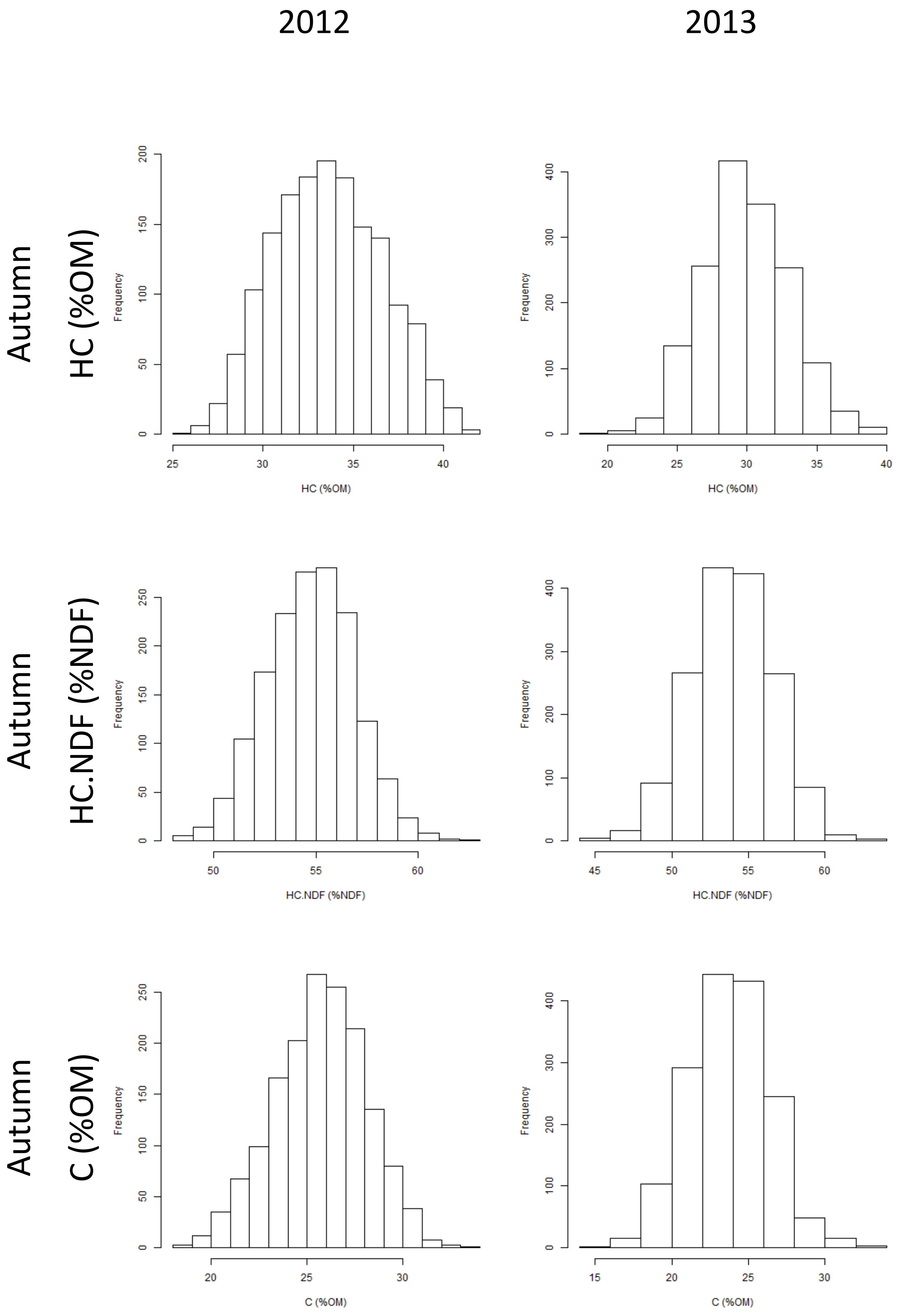
2012
2013
Autumn
HC (%OM)
Autumn
HC.NDF (%NDF)
Autumn
C (%OM)
Frequency
HC (%OM)
HC.NDF (%NDF)
C (%OM)

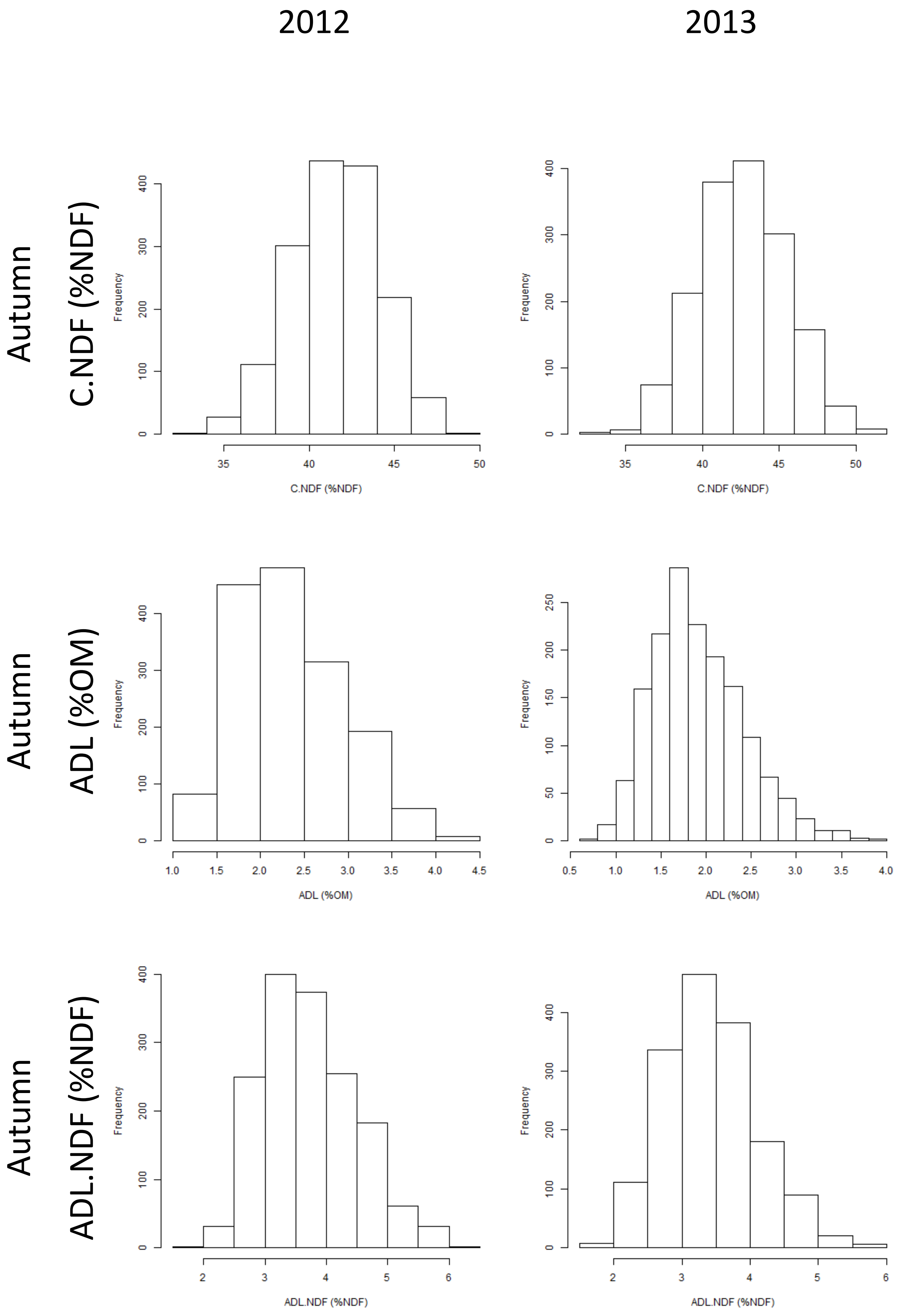
2012
2013
Autumn
C.NDF (%NDF)
Autumn
ADL (%OM)
Autumn
ADL.NDF (%NDF)
Frequency
C.NDF (%NDF)
ADL (%OM)
ADL.NDF (%NDF)
