# Supplementary material for: Seasonal Differences in Structural and Genetic Control of Digestibility in Perennial Ryegrass
Source: Front Plant Sci. 2022 Jan 4;12:801145. doi: 10.3389/fpls.2021.801145 (PMC8765707; doi:10.3389/fpls.2021.801145)
Supplement: Supplementary file 13 [file Data_Sheet_1.docx]

# List of Supplementary figures

Supplementary Figure 1: Graphical representation of FastStructure results. Each color represents an identified group.

Supplementary Figure 2: Principal component analysis (PCA) of 580 perennial ryegrass genotypes based on 252 406 SNPs and 5 074 indels. Groups from population structure are indicated by different colours.

Supplementary Figure 3: Histograms of each trait values by season and by year.

Supplementary Figure 4: Spring blades against autumn plant for all studied traits by year.

# List of Supplementary tables

Supplementary Table 1: Description of Plant Material and grouping obtained by fastStructure analysis

Supplementary Table 2: Summary of phenotypic data

Supplementary Table 3: Genotypic data used for GWAS analysis, first column displays genotype number and following columns contain the marker information. Genotype notation is in allele dosage (0, 1, 2).

Supplementary Table 4: Correlations between quality traits and heading date in spring 2012 and 2013.

Supplementary Table 5: Correlations between seasons for each trait (organic matter digestibility (OMD), fiber content (NDF), fiber digestibility (NDFD), hemicellulose (HC), hemicellulose over NDF (HC.NDF), cellulose (C), cellulose over NDF (C.NDF), lignins (ADL) and lignins over NDF (ADL.NDF)), using Spearman method.

Supplementary Table 6: p-values obtained from GWAS study for each combination of trait, season (spring, spring corrected for harvest date and autumn), and year (2012, 2013 and both).

Supplementary Table 7: Coefficient, p-value and variance explained of each identified QTL for each combination of trait, season (spring, spring corrected for harvest date and autumn), and year (2012, 2013 and both).

Supplementary Table 8: Summary of identified genes for each trait and season (spring corrected for harvest date and autumn). Genes identified for OMD, NDFD and HC.NDF in spring corrected for harvest date were highlighted in green and genes identified for OMD, NDFD and ADL.NDF in autumn were highlighted in red.
